# *Ab Initio* Partition Functions and Thermodynamic Quantities for the Molecular Hydrogen Isotopologues

**DOI:** 10.1021/acs.jpca.1c06468

**Published:** 2021-10-06

**Authors:** José Zúñiga, Adolfo Bastida, Alberto Requena, Javier Cerezo

**Affiliations:** † Departamento de Química Física, Universidad de Murcia, 30100 Murcia, Spain; ‡ Departamento de Química, Universidad Autónoma de Madrid, 28049 Madrid, Spain

## Abstract

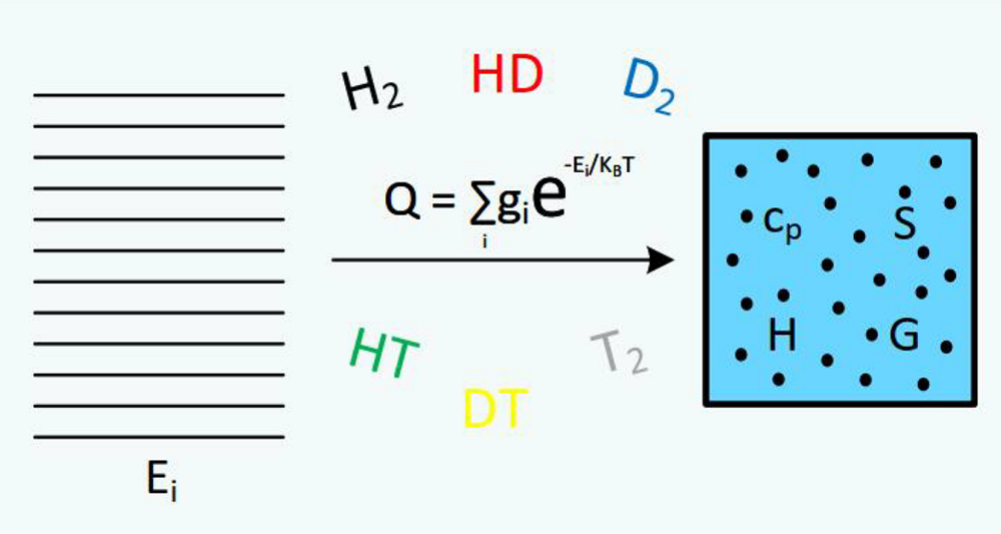

In this work, we calculate the partition functions and thermodynamic quantities of molecular hydrogen isotopologues using the rovibrational energy levels provided by the highly accurate *ab initio* adiabatic potential energy functions recently determined by Pachucki and Komasa (Pachucki, K.; Komasa, J. *J. Chem. Phys.***2014**, *141*, 224103). The partition functions are calculated by including all bound energy levels of the isotopologues, up to their dissociation limits, plus the quasi-bound levels lying below the centrifugal potential barriers. For the homonuclear isotopologues, H_2_, D_2_, and T_2_, we also determine the partition functions and thermodynamic quantities of the normal mixtures using the statistical treatment recently proposed by Colonna et al. (Colonna, G.; D’Angola, A.; Capitelli, M. *Int. J. Hydrogen Energy***2012**, *37*, 9656) based on the definition of the partition function of the mixture, which avoids inconsistencies in the values of the thermodynamic quantities depending directly on the internal partition function, in the high-temperature limit.

## 1 Introduction

There is no doubt that molecular hydrogen is one of the chemicals of the moment. Not surprisingly, it is a key player in the energy transition as an energy storage and transport vector, and as an alternative fuel,^1−5^ and plays an important role in atmospheric and interstellar chemistry.^6−15^ It is therefore essential to accurately characterize their thermodynamic properties, including all their isotopologues.^16,17^

For the main isotopologue H_2_, a good number of studies have been carried out to determine partition functions and thermodynamic quantities of both equilibrium and normal hydrogen.^18−33^ The information available for the remaining isotopologues is, however, much scarcer, not going beyond the stable deuterated isotopologues HD and D_2_,^19,27,29,33^ except for the work by Le Roy et al.^26^ in which they reported the thermodynamic quantities of all isotopologues, although no partition functions.

In this work, we accurately calculate the partition functions and thermodynamic quantities of the six isotopologues of molecular hydrogen in the temperature range from 1 to 10000 K. For this purpose, we use the adiabatic potentials of the isotopologues recently determined by Pachucki and Komasa using high-level *ab initio* methods.^34,35^ Pachucki and Komasa also provided the rovibrational bound energy levels of all isotopologues, up to their dissociation limits, which can be used directly to obtain the partition functions as sums of the exponential energy factors over all the levels. However, they did not calculate the quasi-bound energy levels generated by the centrifugal potential barriers of the isotopologues, whose inclusion in the partition function sums may significantly modify their values and those of the thermodynamic quantities at high temperatures. Because of this, we recalculate the bound energy levels of the Pachucki and Komasa adiabatic potentials using an efficient variational method, and additionally estimate the quasi-bound energy levels also variationally with the accuray required to evaluate the partition functions and thermodynamic quantities.

We have also determined the partition functions and thermodynamic quantities of the normal mixtures of the homonuclear isotopologues H_2_, D_2_, and T_2_, using the rigorous statistical thermodynamic formulation recently developed by Colonna et al.,^30^ which eliminates inconsistencies in the values of thermodynamic quantities depending directly on the partition functions, such as the entropy and the Gibbs free energy, when compared to the equilibrium values at high temperatures.

## 2 Theory

The total partition function of a polyatomic molecule can be expressed as follows:^36,37^
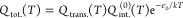
1where *Q*_trans_(*T*) is the translational partition function given by
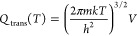
2and *Q*_int._^(0)^(*T*) is the internal partition function in which the energy levels are relative to the ground state energy ε_0_. Assuming that only the electronic ground state is populated, the internal partition function can be written in the form
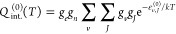
3where *g*_*e*_, *g*_*n*_, *g*_*v*_, and *g*_*J*_ are, respectively, the electronic, nuclear, vibrational, and rotational degeneracy factors, *v* and *J* collectively label the vibrational and rotational quantum numbers of the molecule, and ε_*v,J*_^(0)^ = ε_*v,J*_ – ε_0_.

For diatomic molecules with fundamental electronic state *X*^1^Σ^+^ (heteronuclear) or *X*^1^Σ_*g*_^+^ (homonuclear), such as molecular hydrogen H_2_ and its isotopologues, the vibrational and rotational degeneracy factors are, respectively, *g*_*v*_ = 1 and *g*_*J*_ = 2*J* + 1, and the internal partition function (3) becomes
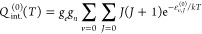
4Moreover, the electronic degeneracy factor *g*_*e*_ is equal to unity (*g*_*e*_ = 1), and the nuclear degeneracy factors are given by
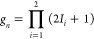
5where *I*_*i*_ are the nuclear spins.

For the homonuclear isotopologues of molecular hydrogen, the coupling between rotational motion and nuclear spin must be taken into account.^36,37^ The internal partition function then splits as follows:
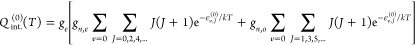
6where *g*_*n*,*e*_ and *g*_*n*,*o*_ are, respectively, the nuclear statistical weights of the even and odd rotational levels, which are given by *g*_*n*,*e*_ = (2*I* + 1)*I* and *g*_*n*,*o*_ = (2*I* + 1)(*I* + 1) if the nuclei are Fermions (semi-integer spin), and *g*_*n*,*e*_ = (2*I* + 1)(*I* + 1) and *g*_*n*,*o*_ = (2*I* + 1)*I* if the nuclei are bosons (integer spin). The values of the nuclear spin factors of the H_2_ isotopologues are included in Table 1. For the H_2_ and T_2_ isotopologues (Fermions), the species with *J* even form para-H_2_ and para-T_2_, and the species with *J* odd form ortho-H_2_ and ortho-T_2_, while for the isotopologue D_2_ (Bosons), the species with *J* even form ortho-D_2_ and the species with *J* odd form para-D_2_.

**Table 1:** Nuclear Spin Properties of the H_2_ Isotopologues^a^

*M*^b^	isotolopogue	*I*_1_	*I*_2_	nuclear system	*g*_*n*_^c^	*g*_*n,e*_^d^	*g*_*n,o*_^e^
2	H_2_	1/2	1/2	Fermions	4	1	3
3	HD	1/2	1		6		
4	HT	1/2	1/2		4		
4	D_2_	1	1	Bosons	9	6	3
5	DT	1	1/2		6		
6	T_2_	1/2	1/2	Fermions	4	1	3

a The nuclear spin values are *I*(H) = 1/2, *I*(D) = 1, and *I*(T) = 1/2.

b Mass number.

c (2*I*_1_ + 1)(2I_2_ + 1),

d (2*I* + 1)*I* (Fermions); (2*I* + 1)(*I* + 1) (bosons),

e (2*I* + 1)(*I* + 1) (Fermions); (2*I* + 1)*I* (bosons),

From the partition functions, we can calculate the thermodynamic quantities of the ideal gas by substituting eq 1 into the general statistical expressions for the thermodynamic quantities. Thus, we obtain the following expressions for the molar energy *E*(*T*), enthalpy *H*(*T*), heat capacity at constant pressure *C*_*P*_(*T*), entropy *S*(*T*), and Gibbs free energy *G*(*T*), in terms of the internal partition function *Q*_int._^(0)^:
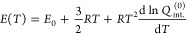
7
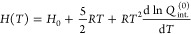
8
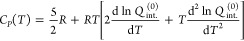
9
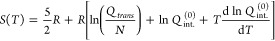
10
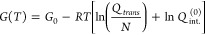
11where *E*_0_ = *H*_0_ = *G*_0_ = *N*_*A*_ε_0_ is the molar energy of the gas at absolute zero temperature.

To facilitate the evaluation of the thermodynamic quantities, it is convenient to use the first and second moments of the internal partition function, *Q*_int._^(0)′^ and *Q*_int._^(0)″^, respectively, which are defined as follows^38^
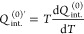
12
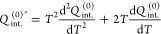
13The expressions for the thermodynamic quantities as a function of the moments are then
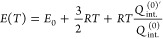
14
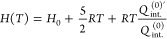
15
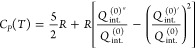
16
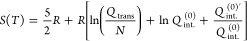
17
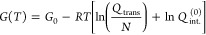
18The interesting thing about the moments *Q*_int._^(0)′^ and *Q*_int._^(0)″^ is that, after using eq 4 for the internal partition function *Q*_int._^(0)^ into their definitions (12) and (13), we get the expressions
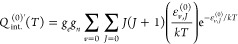
19
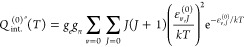
20which allows us to calculate the moments directly from the rovibrational energies of the molecule.

Equation 6 for the internal partition function of homonuclear isotopologues is interpreted as an equilibrium mixture of the species with *J* even and *J* odd rotational quantum numbers, i.e., of the corresponding ortho and para species. And when the populations of the *J* even and *J* odd species are frozen at their equilibrium values at high temperatures, we have the so-called normal mixture of the isotopologue.^36,37^ In principle, it seems logical to calculate the thermodynamic functions of the normal mixture as averages of the functions of the species with *J* even and *J* odd, weighted with their corresponding nuclear statistical factors. This method presents, however, some inconsistencies in the thermodynamic quantities directly depending on the internal partition function, such as the entropy and the Gibbs free energy (eqs 10 and 11), which values for the normal and equilibrium mixtures do not match in the high temperature limit as they should.^18,24,26^ Recently, Colonna et al.^30^ have solved this problem on a rigorous basis by statistically deriving the expression for the internal partition function of the normal, nonequilibrium mixture of species with *J* even and *J* odd.

In the Colonna’s treatment,^30^ the total partition function of the normal mixture is written as follows:
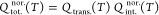
21where *Q*_int._^nor.^(*T*) is the internal partition function of the mixture.^39^ It is then shown that *Q*_int._^nor.^(*T*) can be expressed in terms of the rovibrational partition functions of the species with *J* even and *J* odd, *Q*_*vr*,*e*_(*T*) and *Q*_*vr*,*o*_(*T*), in the form^30^
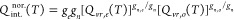
22where
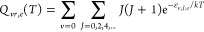
23
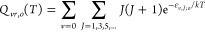
24By extracting the zero-point energy exponentials of the *J* even and *J* odd species from these expressions, we write
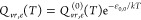
25
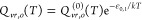
26where
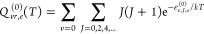
27
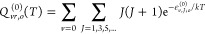
28and ε_*v,J;e*_^(0)^ = ε_*v,J;e*_ – ε_0,0_ and ε_*v,J;o*_^(0)^ = ε_*v,J;o*_ – ε_0,1_. And by using eqs 25 and 26 into eq 22 for *Q*_int._^nor.^(*T*), we obtain
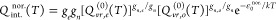
29where ε_0_^nor.^ is the zero-point energy of the normal mixture given by
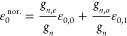
30Introducing now the internal partition functions of the species with *J* even and *J* odd, separately, in the form
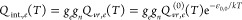
31
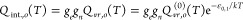
32we get the following expression for the internal partition function of the normal mixture as a function of the internal partition functions of the *J* even and *J* odd species,
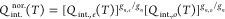
33The expressions for the thermodynamic quantities of the normal mixture are then obtained by substituting the internal partition function of the normal mixture *Q*_int._^nor.^(*T*) into eqs 7–11. Thus, we get for the energy of the normal mixture
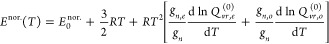
34where *E*_0_^nor.^ = *N*_*A*_ε_0_^nor.^, and it can be easily verified that this expression is equal to the average of the energies of the *J* even and *J* odd species given by
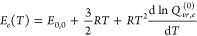
35
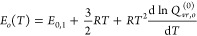
36weighted with the nuclear spin factors, i.e.,
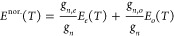
37In an analogous way, the following expressions are obtained for the remaining thermodynamic quantities of the normal mixtures.
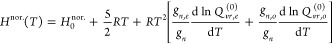
38
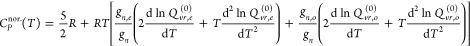
39
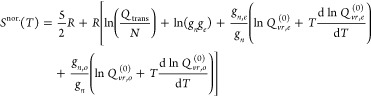
40
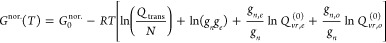
41where *H*_0_^nor.^ = *G*_0_^nor.^ = *E*_0_^nor.^, and all of them can be expressed as averages of the quantities of the *J* even and *J* odd species, weighted with the nuclear spin factors.

We should note that the same is not true when using as internal partition functions of the *J* even and *J* odd species the expressions directly extracted from the total partition function of the mixture at equilibrium (eq 6), i.e.,
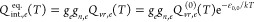
42
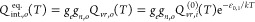
43which differ from eqs 31 and 32 in the nuclear spin factors. In this case, for the thermodynamic quantities which do not contain explicitly the internal partition function, but their derivatives with respect to temperature, such as the energy, the enthalpy, and the heat capacity, their values can still be written as the weighted averages of the thermodynamic quantities of the *J* even and *J* odd species. However, for the thermodynamic quantities depending explicitly on the partition function, such as the entropy and the Gibbs free energy, the resulting weighted averages has to be corrected *ad hoc* by adding nuclear spin dependent terms as follows
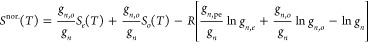
44
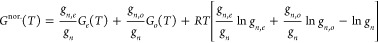
45for these quantities to converge to their equilibrium values at high temperatures, as done by Le Roy et al. in his work.^26^

To thermodynamically characterize the system, it is also convenient to introduce the enthalpy relative to the zero point, or Helmholtz function, *hcf*(*T*), and the reduced Gibbs free energy, or Gibbs enthalpy, *gef*(*T*), which are defined as follows:^27^
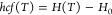
46
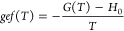
47where *H*_0_ is the molar enthalpy of the gas at the zero point energy of the mixture at equilibrium, ε_0,0_, regardless of whether the mixture is at equilibrium or normal, thus ensuring that the values of hcf(*T*) and gef(*T*) for the normal mixture also converge to their equilibrium values in the high temperature limit.

## 3 Results and Discussion

### 3.1 Energy Levels

To calculate the partition functions of the molecular hydrogen isotopologues, we need their rovibrational energy levels. In this work, we have used the levels of the *ab initio* adiabatic potentials recently determined by Pachucki and Komasa.^34,35^ Pachoucki and Komasa calculated indeed the bound energy levels of the potentials for all isotopologues up to their dissociation limits, which are available in the Supporting Information of ref (34), so they can be used directly to compute the partition functions and thermodynamic quantities of the isotopologues.

As discussed, however, by a number of authors,^19,26,27,30^ it is convenient to include the quasi-bound rovibrational energy levels in the partition functions, especially those lying between the dissociation limit of the isotopologue and the maximum of the centrifugal potential barrier generated by the effective internuclear potential for values of *J* ≠ 0.^40^ Accordingly, we have recalculated variationally the bound energy levels of the Pachucki and Komasa adiabatic potentials, and further determined also variationally the quasi-bound energy levels.

The rovibrational energy levels of the H_2_ isotopologues are obtained by solving the radial Schrödinger equation given by
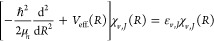
48where *R* is the internuclear distance, μ_*n*_ is the reduced mass of the isotopologue nuclei, ε_*v*,*J*_ and χ_*v*,*J*_(*R*) are, respectively, the rovibrational energy levels and the radial eigenfunctions, and *V*_eff._(*R*) is the effective potential, which is given by the sum of the adiabatic potential *V*_ad._(*R*) and the centrifugal distortion term, i.e.,
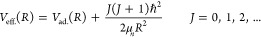
49The adiabatic potential is, in turn, given by the expression
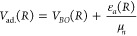
50where *V*_*BO*_(*R*) is the Born–Oppenheimer potential and ε_*a*_(*R*) is the adiabatic diagonal correction term. The expressions for *V*_*BO*_(*R*) and ε_*a*_(*R*) are described in detail by Pachucki and Komasa in refs (34) and (35), and the potential subroutines to calculate their values are available in the Supporting Information of ref (41). In Table, 2 we give the nuclear and reduced masses used for the isotopologues, extracted from CODATA-2018,^42^ from where all the physical constants and conversion factors needed to perform the variational calculations were also taken. In Table 3, we include as well the equilibrium distances *R*_*e*_ and dissociation energies *D*_*e*_ of the isotopologues, along with those of the Born–Oppenheimer (BO) potential common to all of them for comparative purposes.

**Table 2:** Nuclear Masses of the Isotopologues (in ua) Taken from CODATA-2018 and Used to Evaluate the Pachucki and Komasa Adiabatic Potentials (*m*_*p*_ = 1836.15267343 au, *m*_*d*_ = 3670.48296788 ua, *m*_*t*_ = 5496.92153573 ua)

*M*^a^	isotopologue	total mass	reduced mass
2	H_2_	3672.30534686	918.076336715
3	HD	5506.63564131	1223.89922872
4	HT	7333.07420916	1376.39234045
4	D_2_	7340.96593576	1835.24148394
5	DT	9167.40450361	2200.87996169
6	T_2_	10993.8430715	2748.46076799

a Mass number.

**Table 3:** Equilibrium Distances and Dissociation Energies of the H_2_ Isotopologues for the Pachucki and Komasa Adiabatic Potentials^34^

*M*^a^	isotopologue	*R*_*e*_ (Å)	*D*_*e*_ (cm^–1^)	*D*_*o*_ (cm^–1^)	*E*_0,0_ (cm^–1^)
2	H_2_	0.7416254	38298.019151	36118.363713	2179.655438
3	HD	0.7415744	38296.774032	36406.183972	1890.590060
4	HT	0.7415574	38296.360078	36512.608564	1783.751514
4	D_2_	0.7415233	38295.529669	36748.934662	1546.595007
5	DT	0.7415064	38295.115966	36881.884013	1413.231983
6	T_2_	0.7414894	38294.702346	37029.138896	1265.563450
	BO	0.7414212	38293.040738		

a Mass number.

**Figure 1:**
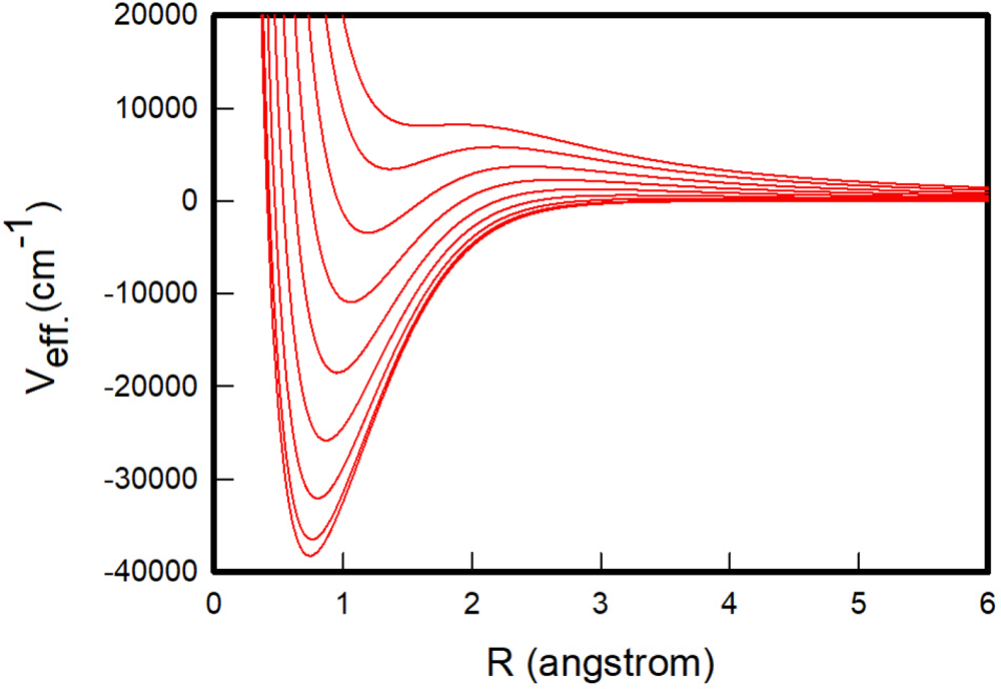
Effective adiabatic Pachucki and Komasa potentials of the H_2_ isotopologue, for the increasing values of the rotational quantum number *J* = 0, 5, 10, 15, 20, 25, 30, 35, and 40.

As noted above, the centrifugal distortion term of the effective potential generates a potential barrier in the attractive curve of the potential function whose position, *R*_*b*_, and height, *V*_*b*_, vary as a function of *J*, as shown in Figure 1 for the main isotopologue H_2_. Also, the depth of the effective potential well, *V*_*o*_, which minimum is located at *R*_*o*_, decreases as *J* increases until the well and the barrier fade away for a given value of *J*, and the effective potential becomes unbounded. By setting the zero energy at the dissociation limit, the rovibrational bound energy levels are those which satisfy *E*_*v*,*J*_ < 0, while the quasi-bound energy levels are those lying between the dissociation limit and the maximum of the barrier, i.e., those satisfying the condition 0 < *E*_*v*,*J*_ < *V*_*b*_(*J*) with their wave functions accumulating most of the probability density in the potential well region. In order to determine the quasi-bound levels, it is therefore necessary to have the *R*_*b*_ and *V*_*b*_ parameters that characterize the centrifugal potential barriers available. Table 4 gives the values of these parameters for the effective potentials of H_2_ depicted in Figure 1, along with those for the minima of these potentials. The minima and maxima of the effective potentials of the six isotopologues of H_2_ for all values of the rotational quantum number *J* are given in the Supporting Information.

**Table 4:** Minima and Maxima of the Effective Potentials of the Main Isotopologue H_2_

*J*	*R*_*o*_ (Å)	*V*_*o*_ (cm^–1^)	*R*_*b*_ (Å)	*V*_*b*_ (cm^–1^)
0	0.7416254	–38298.019151	0.000000	0.000000
5	0.7583952	–36512.859489	4.061847	67.20030
10	0.8012586	–32108.601036	3.579876	226.9986
15	0.8667136	–25848.197588	3.190941	613.3744
20	0.9521347	–18553.181653	2.898881	1269.863
25	1.057507	–10909.635729	2.652182	2276.197
30	1.187589	–3439.9851650	2.422940	3739.023
35	1.360698	3428.2573833	2.180216	5829.746
39	1.610548	8120.2806688	1.890646	8226.017

We have solved the radial Schrödinger equation (48) with the HEG variational method,^43,44^ using the eigenfunctions of the particle in a box as the basis functions set. After several convergence tries, we decided to use a box with boundaries [0.01, 22] Å, 300 basis functions to perform the variational calculations, and 600 quadrature points to evaluate the matrix elements of the effective potentials. This basis set guarantees convergence in the first eight significant digits of the bound energy levels of all isotopologues, similar to that obtained by Pachucki and Komasa in their calculations.^34^ There are some residual differences between our results and those by Pachucki and Komasa, likely due to the slightly different values used for the nuclear masses, physical constants and conversion factors. Nevertheless, the values obtained in our calculations for the ground states energies of the isotopologues, ε_0,0_, which directly provide the chemical dissociation energies *D*_0_ = −ε_0,0_, agree in the first six significant digits with those of Pachucki and Komasa. The values of *D*_0_ are also given in Table 3, along with their differences with the dissociation energies *D*_*e*_, which provides the zero point energies relative to the minima of the adiabatic potentials. To check that the residual numerical differences between our bound energy levels and those by Pachucki and Komasa do not affect the determination of the partition functions of the isotopologues, we have calculated them using both sets of energy levels finding practically no differences.

As far as the quasi-bound energy levels are concerned, their rigorous determination requires methods that guarantee the fulfillment of the boundary conditions of these states, embedded in the continuum of energy levels,^45^ and provide their half-lives accounting for the predissociation of the molecule. Nevertheless, an easy way of estimating the quasi-bound levels consists of leveling the centrifugal potential barriers beyond their maxima, i.e., modifying the effective potentials so that *V*_*eff*._(*R*) = *V*_*b*_ for *R* ≥ *R*_*b*_. Thus, the effective potentials become bounded potentials, with dissociation limits given by the maxima of the centrifugal barriers, and the variational HEG method can be additionally used to determine, in an approximate way, the quasi-bound energy levels. This method is equivalent to estimating the quasi-bound energy levels by extrapolation of the Dunham series obtained by fitting the rovibrational spectral lines of the molecules measured experimentally.^18,27,30^

The variational calculations performed for the main isotopologue of H_2_ by smoothing the centrifugal potential barriers give a total of 49 quasi-bound energy levels, to be added to the 301 bound levels of the isotopologue, what represents about 16% more energy levels in the calculation of the partition function. For the remaining isotopologues, the ratios of quasi-bound to bound levels are similar. Table 5 includes the numbers of bound and quasi-bound levels, along with the total number of levels for each isotopologue, which logically increase with the mass of the isotopologue. The values of the quasi-bound energy levels obtained for all isotopologues are given in the Supporting Information.

**Table 5:** Increasing Number of Rovibrational Energy Levels of the H_2_ Isotopologues Calculated Using the Pachucki and Komasa Adiabatic Potentials Including the Quasi-Bound Energy Levels

*M*^a^	isotopologue	bound	quasi-bound	total
2	H_2_	301	49	350
3	HD	400	67	467
4	HT	449	78	527
4	D_2_	601	99	700
5	DT	720	118	838
6	T_2_	897	150	1047

a Mass number.

### 3.2 Partition Functions

As already indicated above, a great effort has been made to calculate the partition function of the main isotopologue H_2_.^20−23,25,28−32^ Most of these works have been recently reviewed by Popovas and Jorgensen,^31^ making a systematic study of the different approaches that can be used to calculate the partition functions, from the most elementary one based on the harmonic oscillator-rigid rotator model, to the most rigorous one consisting of summing the Boltzmann factors over the rovibrational energy levels provided, in their case, by empirical high-order Dunham expansions. The partition functions of the H_2_ and HD isotopologues deposited in the HITRAN database are also available,^29,33^ as calculated by summation over the rovibrational energy levels also calculated theoretically by Pachucki and co-workers, although coming from earlier works by these authors^46,47^ than that used in the present article.^34^ Let us see then first how these most recently determined partition functions for the H_2_ and HD isotopologues compare with ours.

In Table 6, we include the internal partition functions of the main isotopologue H_2_ taken from the HITRAN database, those calculated by Popovas and Jorgensen (PJ), and those obtained in this work using the rovibrational bound energy levels of the Pachucki and Komasa adiabatic potentials and adding the quasi-bound levels. The HITRAN partition functions and the Popovas–Jorgensen ones were calculated using only the bound energy levels, so we have to compare them with our partition functions calculated the same way.

**Table 6:** Internal Partition Functions *Q*_int._^(0)^(*T*) of H_2_ Extracted from the HITRAN Database and Calculated by Popovas and Jorgensen (PJ) from Dunham Expansions of the Rovibrational Energy Levels of Electronic States and by Summation over the Rovibrational Energy Levels Extracted from the Adiabatic Pachoucki and Komasa Potentials Including Only the Bound Levels (Present-bl) and Adding the Quasi-Bound Levels (Present-qbl)

*T* (K)	HITRAN^a^	Δ*Q*_HITRAN_^b^	PJ^c^	Δ*Q*_PJ_^d^	present-bl^e^	present-qbl^f^	Δ*Q*_bl_^g^
10	1.000000	0.000	1.000000	0.000	1.000000	1.000000	0.000
50	1.297714	0.004	1.297887	0.018	1.297656	1.297656	0.000
100	2.667730	0.006	2.668230	0.025	2.667561	2.667561	0.000
150	4.079808	0.006	4.080472	0.022	4.079578	4.079578	0.000
200	5.361676	0.005	5.362463	0.020	5.361394	5.361394	0.000
250	6.575840	0.005	6.576747	0.019	6.575500	6.575500	0.000
300	7.766034	0.005	7.767062	0.018	7.765630	7.765630	0.000
400	10.13487	0.005	10.13613	0.018	10.13434	10.13434	0.000
500	12.51071	0.005	12.51218	0.017	12.51006	12.51006	0.000
600	14.89709	0.005	14.89894	0.016	14.89653	14.89653	0.000
700	17.29468	–0.002	17.29763	0.015	17.29496	17.29496	0.000
800	19.70520	–0.015	19.71113	0.015	19.70822	19.70822	0.000
900	22.13171	–0.042	22.14418	0.014	22.14104	22.14104	0.000
1000	24.57844	–0.086	24.60293	0.014	24.59958	24.59958	0.000
2000	51.36081	–1.331	52.05989	0.012	52.05356	52.05356	0.000
3000	85.63429	–2.516	87.85665	0.014	87.84444	87.84450	0.000
4000	130.4869	–2.989	134.5280	0.016	134.5068	134.5128	0.004
5000	187.9761	–3.072	193.9682	0.017	193.9345	194.0253	0.047
6000	259.7835	–3.030	267.9527	0.019	267.9022	268.4588	0.207
7000			357.7921	0.020	357.7197	359.7595	0.567
8000			463.9564	0.021	463.8571	469.2692	1.153
9000			585.9934	0.023	585.8590	597.4331	1.937
10000			722.6994	0.026	722.5085	743.7856	2.861

a Reference (33).

b Δ*Q*_HITRAN_ = 100 × (*Q*_HITRAN_ – *Q*_Present-bl_)/*Q*_Present-bl_.

c Reference (31).

d Δ*Q*_PJ_ = 100 × (*Q*_PJ_ – *Q*_Present-bl_)/*Q*_Present-bl_.

e Including only the bound-levels (bl).

f Adding the quasi-bound levels (qbl).

g Δ*Q*_bl_ = 100 × (*Q*_qbl_ – *Q*_Present-bl_)/*Q*_Present-qbl_.

As observed in Table 6, the HITRAN partition functions are quite accurate at low temperatures, but increasingly differ from our partition functions for temperatures higher than 1000 K, reaching relative errors of 3% in the highest range of temperatures, from 3000 to 6000 K, spanned by HITRAN database. The partition functions calculated by Popovas and Jorgensen using an accurate empirical Dunham expansion for the rovibrational energies, compare, however, much better with ours, providing values that practically reproduce our partition functions up to 10000 K, with relative errors always below 0.030%.

Let us see now what happens when we include the quasi-bound energy levels in the calculations. The resulting partition functions thus obtained are included in the penultimate column of Table 6. As observed, their values agree, in all the significant digits given, with the partition functions calculated using solely the bound levels up to 2000 K, and from here on, the partition functions including the quasi-bound levels start to gradually increase with respect to those containing only the bound levels, as expected when adding more terms to the Boltzmann summation, until reaching a deviation of about 20 absolute units at the highest temperature of 10000 K, which represents a relative error of 2.861%. The quasi-bound levels start to noticeably modify the H_2_ partition functions at 2000–3000 K, and their effect becomes more and more pronounced as the temperature rises, becoming quite significant from 5000 to 6000 K onward.

Al for the HD isotopologue, we only have for comparison the partition functions deposited in the HITRAN^29,33^ database. Their values, along with ours extracted from the Pachucki and Komasa potential, are shown in Table 7. Interestingly, for this isotopologue the HITRAN partition function remains quite accurate up to 6000 K, with relative errors that, although slowly increasing, keep practically below 0.02% in that temperature range. It seems to be that the *ab initio* rovibrational energy levels used to calculate the HITRAN partition function of the deuterated isotopologue HD were of better quality than those used to calculate the HITRAN partition function of the main isotopologue H_2_.^46,47^ When including the quasi-bound levels in our calculations, again the HD partition functions start to increase above those obtained using only the bound levels at 2000 K, and the differences grow until reaching about 93 absolute units at the highest temperature of 10000 K, representing a relative error of 2.834% similar to that obtained for the main isotopologue H_2_.

**Table 7:** Internal Partition Functions *Q*_int*.*_^(0)^(*T*) of HD Extracted from the HITRAN Database and Calculated by Summation over the Rovibrational Energy Levels Extracted from the Adiabatic Pachoucki and Komasa Potentials Including Only the Bound Levels (Present-bl) and Adding the Quasi-Bound Levels (Present-qbl)

*T* (K)	HITRAN^a^	Δ*Q*_HITRAN_^b^	present-bl^c^	present-qbl^d^	Δ*Q*_bl_^e^
10	6.000048	0.000	6.000048	6.000048	0.000
50	7.394774	0.004	7.394503	7.394503	0.000
100	11.64890	0.006	11.64823	11.64823	0.000
150	16.22985	0.006	16.22882	16.22882	0.000
200	20.87753	0.007	20.87615	20.87615	0.000
250	25.55482	0.007	25.55309	25.55309	0.000
300	30.25068	0.007	30.24860	30.24860	0.000
400	39.68262	0.007	39.67983	39.67983	0.000
500	49.15955	0.007	49.15603	49.15603	0.000
600	58.68270	0.007	58.67846	58.67846	0.000
700	68.26512	0.007	68.26011	68.26011	0.000
800	77.93091	0.007	77.92509	77.92509	0.000
900	87.71234	0.008	87.70562	87.70562	0.000
1000	97.64579	0.008	97.63808	97.63808	0.000
2000	212.1631	0.011	212.1389	212.1389	0.000
3000	366.8685	0.015	366.8144	366.8147	0.000
4000	571.7602	0.017	571.6614	571.6867	0.004
5000	834.9470	0.019	834.7860	835.1725	0.046
6000	1164.354	0.021	1164.111	1166.506	0.205
7000			1565.636	1574.475	0.561
8000			2041.654	2065.242	1.142
9000			2590.297	2640.970	1.919
10000			3206.224	3299.722	2.833

a Reference (33).

b Δ*Q*_HITRAN_ = 100 × (*Q*_HITRAN_ – *Q*_Present-bl_)/*Q*_Present-bl_.

c Including only the bound-levels (bl).

d Adding the quasi-bound levels (qbl).

e Δ*Q*_bl_ = 100 × (*Q*_qbl_ – *Q*_Present-bl_)/*Q*_Present-qbl_.

**Figure 2:**
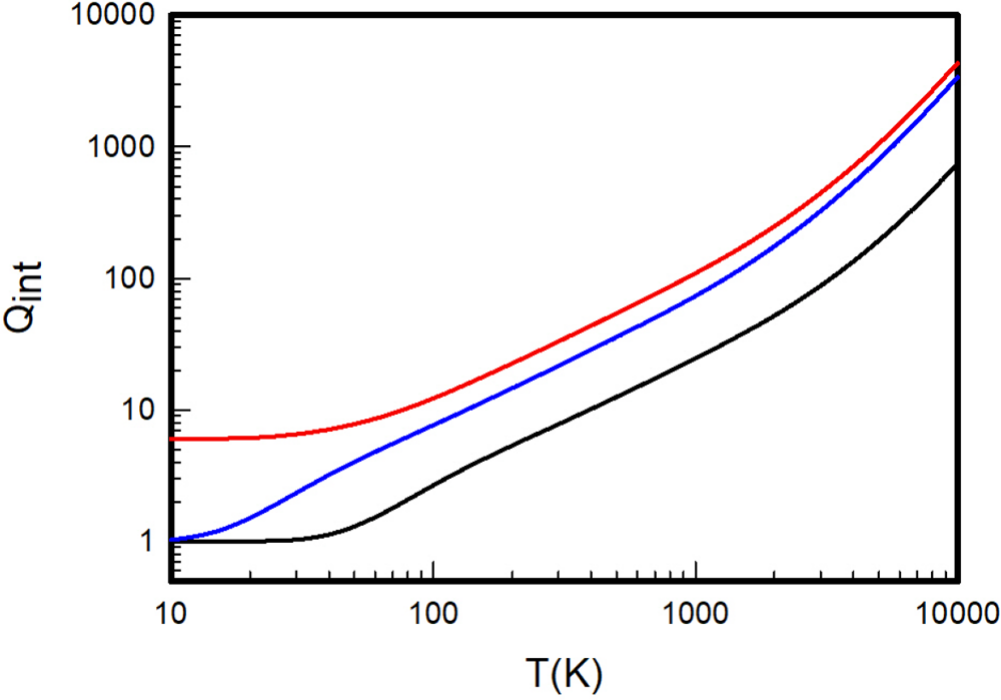
Internal partition functions of H_2_ (black), D_2_ (red), and T_2_ (blue) calculated by summation over the *ab initio* rovibrational energy levels extracted from the adiabatic Pachoucki and Komasa potentials.

**Figure 3:**
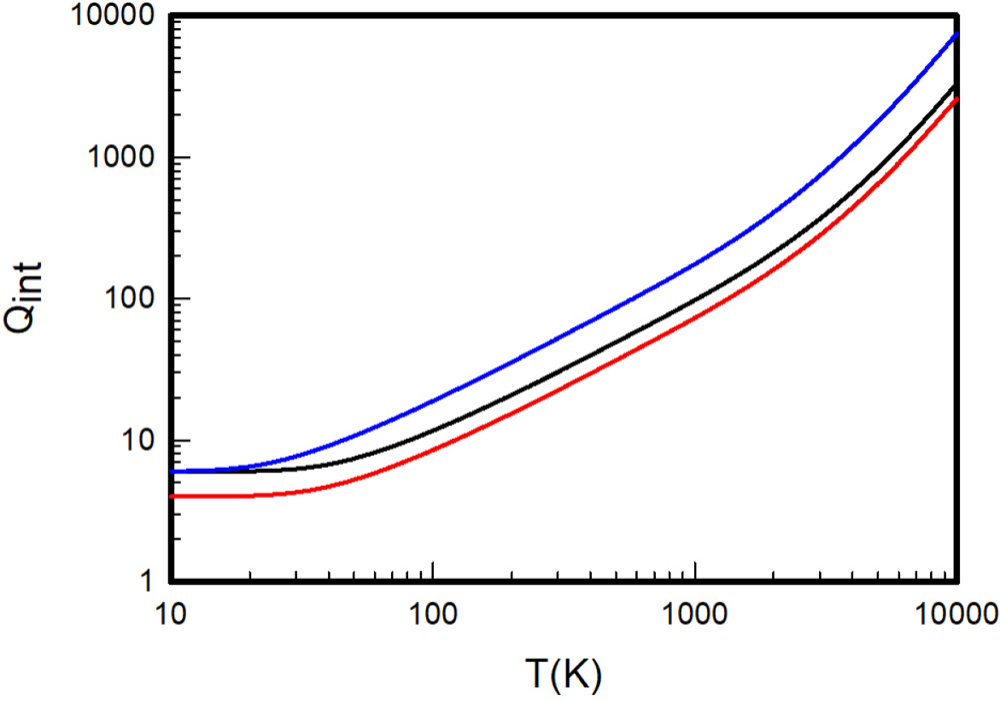
Internal partition functions of HD (black), HT (red), and DT (blue) calculated by summation over the *ab initio* rovibrational energy levels extracted from the adiabatic Pachoucki and Komasa potentials.

For the remaining isotopologues, HT, D_2_, DT, and T_2_, as far as we know there are no prior partition functions to compare with. In any case, the *ab initio* partition functions calculated for them including the quasi-bound levels, whose values are given in Table 8 along with those of the H_2_ and HD isotopologues, are expected to be as accurate as the partition functions obtained for these two isotopologues. For the remaining isotopologues HT, D_2_, DT, and T_2_, the differences between the partition functions calculated using only the bound energy levels and those calculated adding the quasi-bound levels follow the same pattern as that observed for the isotopologues H_2_ and HD; i.e., they become noticeable at 5000–6000 K and reach relative errors of about 2.8% at 10000 K. The partition functions of all isotopologues versus temperature up to 10000 K are depicted in Figures 2 and 3 for a better visualization of them.

**Table 8:** Internal Partition Functions *Q*_int*.*_^(0)^(*T*) of H_2_ Isotopologues Calculated by Summation over the Rovibrational Energy Levels of the Adiabatic Pachoucki and Komasa Potentials Including the Quasi-Bound States

*T* (K)	*Q*(H_2_)	*Q*(HD)	*Q*(D_2_)	*Q*(HT)	*Q*(DT)	*Q*(T_2_)
10	1.000000	6.000048	6.001655	4.000130	6.013645	1.028241
50	1.297656	7.394503	7.785411	5.240706	10.69046	4.020855
100	2.667561	11.64823	12.22288	8.508528	18.87590	7.647912
150	4.079578	16.22882	17.32794	11.95696	27.20476	11.12462
200	5.361394	20.87615	22.55454	15.44495	35.57670	14.60757
250	6.575500	25.55309	27.80967	18.95150	43.97439	18.09935
300	7.765630	30.24860	33.07993	22.47026	52.39246	21.59888
400	10.13434	39.67983	43.65698	29.53554	69.28503	28.62246
500	12.51006	49.15603	54.28503	36.63362	86.26924	35.69493
600	14.89653	58.67846	64.98552	43.76842	103.4027	42.85611
700	17.29496	68.26011	75.80039	50.95397	120.7792	50.16042
800	19.70822	77.92509	86.78502	58.21326	138.5100	57.66484
900	22.14104	87.70562	97.99920	65.57500	156.7055	65.42120
1000	24.59958	97.63808	109.5004	73.07000	175.4655	73.47327
2000	52.05356	212.1389	248.6920	160.6778	407.5017	175.6482
3000	87.84450	366.8147	445.3189	280.6550	741.1252	325.4635
4000	134.5128	571.6867	710.6683	440.4920	1194.645	530.7831
5000	194.0253	835.1725	1055.486	646.7182	1786.428	799.9507
6000	268.4588	1166.506	1492.032	906.6064	2537.708	1142.736
7000	359.7595	1574.475	2032.187	1227.146	3469.241	1568.769
8000	469.2692	2065.242	2684.470	1613.304	4596.107	2085.131
9000	597.4331	2640.970	3452.125	2066.918	5924.310	2694.758
10000	743.7856	3299.722	4332.882	2586.578	7450.282	3396.169

Let us finally consider the partition functions of the normal mixtures of the homonuclear isotopologues H_2_, D_2_, and T_2_. For the main isotopologue H_2_, we have to compare with the empirical partition functions obtained by Popovas and Jorgensen^31^ using only the bound levels, and those obtained also empirically by Colonna et al.^30^ using the H_2_ spectroscopic constants extracted from the NIST database^48^ to calculate the rovibrational energy levels including the quasi-bound levels as well. Both Popovas and Jorgensen, and Colonna et al. use also in their calculations the rovibrational energy levels of the excited electronic states of the H_2_ molecule, although the effect of them starts to be important at 10000 K,^31^ which is the maximum temperature considered in this work.

In Table 9, we give the normal H_2_ isotopologue partition functions calculated by Popovas and Jorgensen and by Colonna et al., along with ours, and the corresponding relative errors. As we see, the empirical partition functions of Popovas and Jorgensen, agree very well with our *ab initio* partitions functions up to the temperature limit of 10000 K, with differences similar to those obtained for the equilibrium mixture of the ortho and para species (see Table 6). On the other hand, the partition functions by Colonna et al. agree well with ours up to ∼1000 K and deviate more and more for higher temperatures until reaching relative errors of about 1%. These discrepancies are likely due to the lower accuracy of the empirical rovibrational energy levels used by Colonna et al. to calculate the partition functions, as opposed to the much more accurate Dunham expansion employed by Popovas and Jorgensen.

**Table 9:** Internal Partition Functions *Q*_int*.*_^(0)^(*T*) of H_2_ for Normal Mixtures Calculated by Colonna et al. and Popovas and Jorgensen (PJ) and by Summation over the Rovibrational Energy Levels Extracted from the Adiabatic Pachoucki and Komasa Potentials Including Only the Bound Levels (Present-bl) and Adding the Quasi-Bound Levels (Present-qbl)

*T* (K)	PJ^a^	Δ*Q*_PJ_^b^	Colonna^c^	Δ*Q*_C_^d^	present-bl	present-qbl	Δ*Q*_bl_^e^
10	9.118028	0.000	9.118080	0.001	9.118028	9.118028	0.000
50	9.118454	0.000	9.118480	0.000	9.118454	9.118454	0.000
100	9.190341	0.001	9.190280	0.000	9.190265	9.190265	0.000
150	9.536879	0.003	9.536560	–0.000	9.536586	9.536586	0.000
200	10.15881	0.005	10.15820	–0.001	10.15826	10.15826	0.000
250	10.96661	0.007	10.96576	–0.000	10.96581	10.96581	0.000
300	11.89362	0.009	11.89252	–0.001	11.89258	11.89258	0.000
400	13.95293	0.010	13.95140	–0.001	13.95148	13.95148	0.000
500	16.15726	0.011	16.15536	–0.001	16.15547	16.15547	0.000
600	18.43674	0.011	18.43460	–0.000	18.43466	18.43466	0.000
700	20.76334	0.011	20.76100	0.000	20.76099	20.76099	0.000
800	23.12641	0.011	23.12400	0.001	23.12384	23.12384	0.000
900	25.52382	0.011	25.52140	0.001	25.52103	25.52103	0.000
1000	27.95788	0.011	27.95560	0.003	27.95488	27.95488	0.000
2000	55.49603	0.011	55.50800	0.032	55.49009	55.49009	0.000
3000	91.68123	0.013	91.78800	0.129	91.66938	91.66945	0.000
4000	138.8967	0.015	139.4188	0.386	138.8758	138.8821	0.004
5000	198.9913	0.017	200.6188	0.788	198.9579	199.0510	0.047
6000	273.7228	0.018	277.3508	1.134	273.6725	274.2412	0.207
7000	364.3861	0.020	370.8300	1.211	364.3139	366.3912	0.567
8000	471.4296	0.021	481.2400	0.925	471.3304	476.8296	1.153
9000	594.3760	0.023	607.7880	0.298	594.2416	605.9811	1.937
10000	731.9970	0.026	748.9760	–0.581	731.8058	753.3560	2.861

a Reference (31).

b Δ*Q*_PJ_ = 100 × (*Q*_PJ_ – *Q*_Present-bl_)/*Q*_Present-bl_.

c Reference (30).

d Δ*Q*_C_ = 100 × (*Q*_C_ – *Q*_Present-qbl_)/*Q*_Present-qbl_.

e Δ*Q*_bl_ = 100 × (*Q*_qbl_ – *Q*_Present-bl_)/*Q*_Present-qbl_.

**Figure 4:**
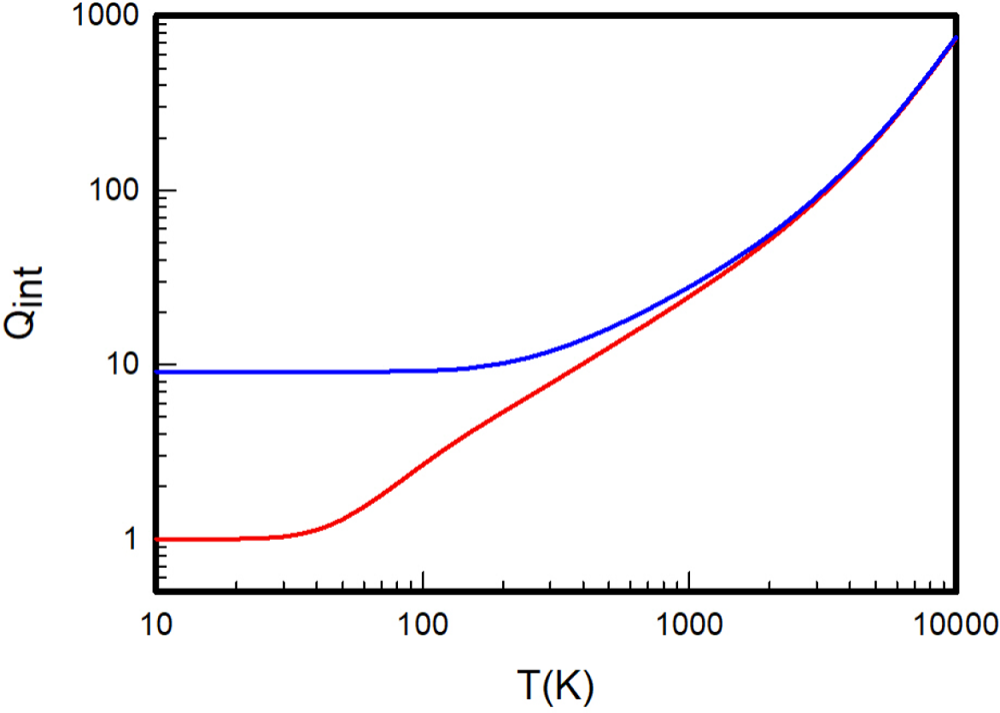
Internal partition functions of equilibrium (red) and normal (blue) H_2_ calculated by summation over the *ab initio* rovibrational energy levels extracted from the adiabatic Pachoucki and Komasa potentials.

**Figure 5:**
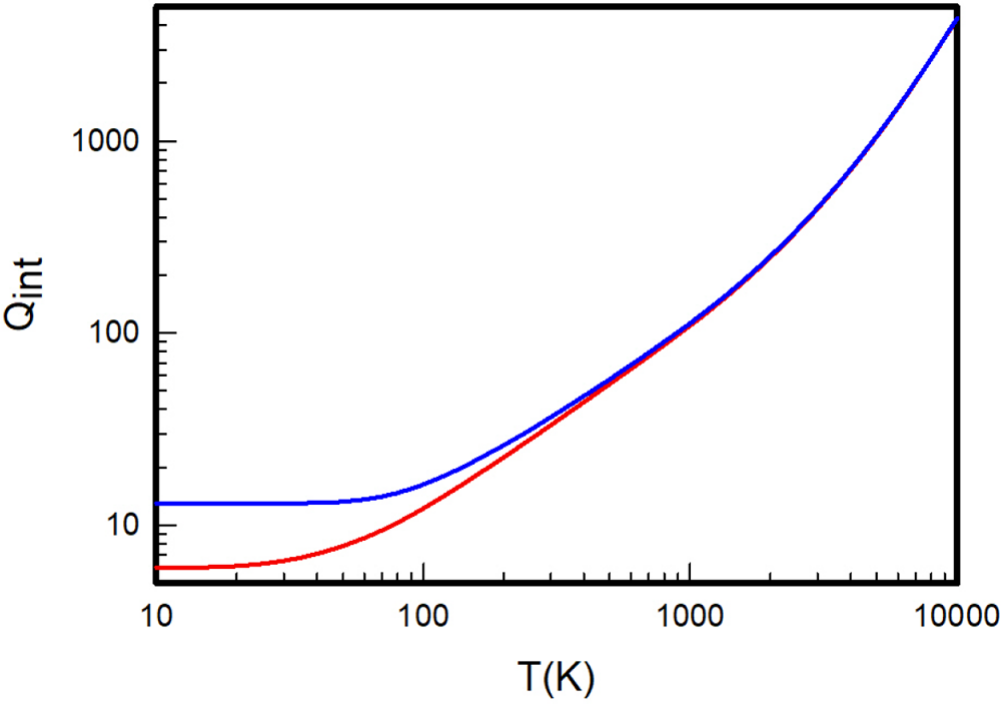
Internal partition functions of equilibrium (red) and normal (blue) D_2_ calculated by summation over the *ab initio* rovibrational energy levels extracted from the adiabatic Pachoucki and Komasa potentials.

**Figure 6:**
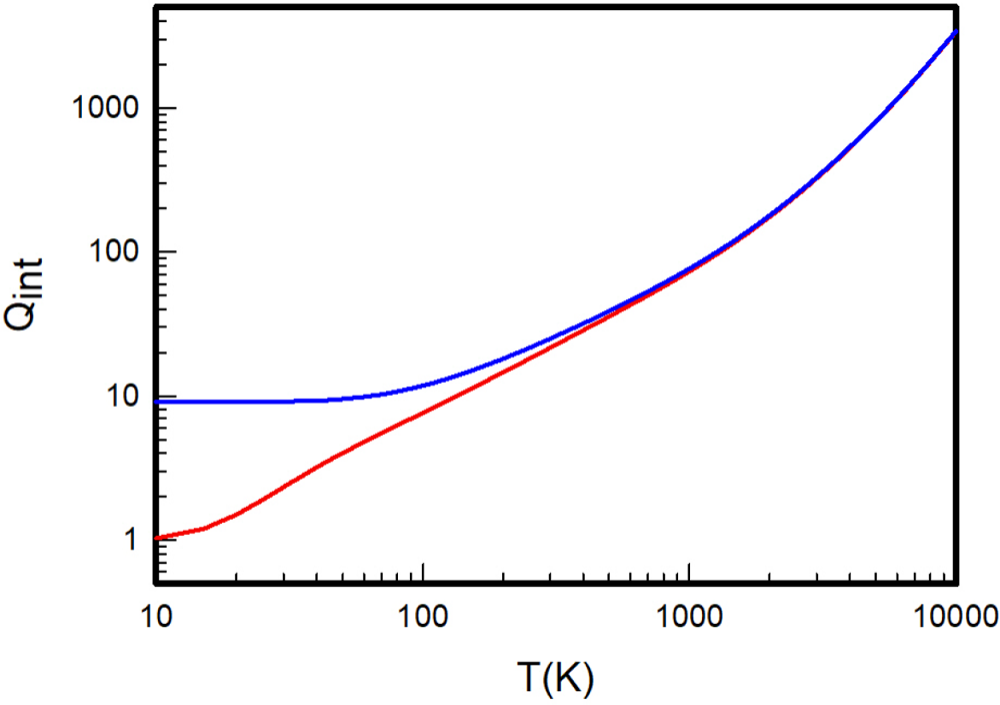
Internal partition functions of equilibrium (red) and normal (blue) T_2_ calculated by summation over the *ab initio* rovibrational energy levels extracted from the adiabatic Pachoucki and Komasa potentials.

The proven good quality of our *ab initio* partition functions for normal H_2_ somehow guarantees the accuracy of the partition functions of the normal D_2_ and T_2_ isotopologues extracted from the Pachucki and Komasa adiabatic potentials. The *ab initio* partition functions of the equilibrium and normal mixtures of the three homonuclear isotopologues H_2_, D_2_, and T_2_ are given in Table 10, and depicted respectively in Figures 4, 5, and 6 to clearly appreciate their noticeable differences at low temperatures.

**Table 10:** Internal Partition Functions *Q*_int*.*_^(0)^(*T*) of Homonuclear H_2_ Isotopologues for Equilibrium and Normal Mixtures Calculated by Summation over the Rovibrational Energy Levels Computed from the Adiabatic Pachoucki and Komasa Potentials Including the Quasi-Bound States

	H_2_		D_2_		T_2_	
*T* (K)	equilibrium	normal	equilibrium	normal	equilibrium	normal
10	1.000000	9.118028	6.001655	12.98025	1.028241	9.118029
50	1.297656	9.118454	7.785411	13.23124	4.020855	9.511511
100	2.667561	9.190265	12.22288	16.27621	7.647912	11.78405
150	4.079578	9.536586	17.32794	20.97764	11.12462	14.84067
200	5.361394	10.15826	22.55454	26.03105	14.60757	18.13234
250	6.575500	10.96581	27.80967	31.18904	18.09935	21.51611
300	7.765630	11.89258	33.07993	36.39735	21.59888	24.94683
400	10.13434	13.95148	43.65698	46.90105	28.62246	31.88930
500	12.51006	16.15547	54.28503	57.48879	35.69493	38.91858
600	14.89653	18.43466	64.98552	68.16622	42.85611	46.05797
700	17.29496	20.76099	75.80039	78.96949	50.16042	53.35595
800	19.70822	23.12384	86.78502	89.95167	57.66484	60.86675
900	22.14104	25.52103	97.99920	101.1714	65.42120	68.64041
1000	24.59958	27.95488	109.5004	112.6853	73.47327	76.71929
2000	52.05356	55.49009	248.6920	252.2827	175.6482	179.4863
3000	87.84450	91.66945	445.3189	449.5952	325.4635	330.1876
4000	134.5128	138.8821	710.6683	715.7805	530.7831	536.5508
5000	194.0253	199.0510	1055.486	1061.556	799.9507	806.8973
6000	268.4588	274.2421	1492.032	1499.179	1142.736	1151.000
7000	359.7595	366.3912	2032.187	2040.528	1568.769	1578.487
8000	469.2092	476.8296	2684.470	2694.108	2085.131	2096.429
9000	597.4331	605.9811	3452.125	3463.139	2694.758	2707.733
10000	743.7856	753.3560	4332.882	4345.319	3396.169	3410.883

### 3.3 Thermodynamic Quantities

As far as the thermodynamic quantities of the molecular hydrogen isotopologues are concerned, we have focused, following the structure of the JANAF database,^27^ on four of them, the heat capacity at constant pressure *C*_*P*_(*T*), the entropy *S*(*T*), the enthalpy relative to the zero point *hcf*(*T*) = *H*(*T*) – *H*_0_, and the reduced Gibbs free energy *gef*(*T*) = −(*G*(*T*) – *H*_0_)/*T*, from which the rest of thermodynamic quantities can be easily derived.^26^

For comparison purposes, we have the values of these thermodynamic quantities for the main isotopologue H_2_ calculated empirically by Popovas and Jorgensen,^31^ the thermodynamic quantities deposited in the JANAF database for the H_2_, HD, and D_2_ isotopologues,^27^ and the *ab initio* values calculated by Le Roy et al.^26^ for the six isotopologues. We should note that all these results are not directly comparable, since each author employs a different convention to give the thermodynamic quantities. Concretely, in the JANAF database,^27^ the room temperature *T*_*r*_ = 298.15 K is used as the reference temperature, instead of absolute zero, and the nuclear spin factors are normalized to unity. Popovas and Jorgensen^31^ use absolute zero as the reference temperature, and maintain the normalization of the nuclear spin factors to unity. And Le Roy et al.^26^ also use absolute zero as the reference temperature employing the real values of the nuclear spin factors, which is the form in which we give the thermodynamic quantities in this work. Nevertheless, we transform our thermodynamic quantities according to the conventions used by each author in order to make the proper comparisons. The calculations by Popovas and Jorgensen are made using only the bound energy levels, as already noticed, while those by Le Roy and the JANAF database incorporate the quasi-bound energy levels.

Let us start with the heat capacity *C*_*P*_(*T*), which is the thermodynamics quantity most sensitive to the partition function, since it depends on the second derivative of the partition function (eq 9). In Table 11 we compare the heat capacities obtained in this work for the main isotopologue H_2_ with those determined by Popovas and Jorgensen, those deposited in the JANAF database, and the ones calculated by Le Roy et al. The relative percentage errors of the heat capacity values with respect to our values are given in parentheses in the table.

**Table 11:** Values of *C*_*P*_^°^ (J K^–1^ mol^–1^) for the Main Isotoplogue H_2_ Calculated Using Different Methods Including Quasi-Bound Levels^a^

*T* (K)	Popovas-Jorgensen^b^	present-bl	JANAF^c^	Le Roy^d^	present-qbl
10	20.7870(0.000)	20.7870		20.7871(0.001)	20.7870
50	37.9699(−0.001)	37.9703		37.9704(0.000)	37.9703
100	28.1511(−0.009)	28.1535	28.154(0.000)	28.1532(−0.001)	28.1535
150	26.5557(−0.001)	26.5559		26.5561(0.001)	26.5559
200	27.4479(−0.002)	27.4475	27.447(−0.004)	27.4478(0.001)	27.4475
250	28.3449(0.001)	28.3446	28.344(−0.004)	28.3449(0.001)	28.3446
300	28.8489(−0.000)	28.8490	28.849(0.000)	28.8492(0.001)	28.8490
400	29.1809(−0.002)	29.1814	29.181(0.000)	29.1816(0.001)	29.1814
500	29.2593(−0.002)	29.2600	29.260(0.000)	29.2602(0.001)	29.2600
600	29.3261(−0.002)	29.3268	29.327(0.000)	29.3271(0.001)	29.3268
700	29.4397(−0.002)	29.4404	29.441(0.003)	29.4407(0.001)	29.4404
800	29.6227(−0.002)	29.6232	29.624(0.003)	29.6237(0.002)	29.6232
900	29.8799(−0.001)	29.8803	29.881(0.003)	29.8810(0.002)	29.8803
1000	30.2037(−0.001)	30.2039	30.205(0.003)	30.2048(0.003)	30.2039
2000	34.2780(0.003)	34.2769	34.280(0.009)	34.2784(0.004)	34.2769
3000	37.0772(0.003)	37.0761	37.087(0.024)	37.0791(0.004)	37.0778
4000	39.0432(0.001)	39.0428	39.116(0.051)	39.0946(−0.002)	39.0955
5000	40.5076(0.006)	40.5053	40.829(0.015)	40.8116(−0.027)	40.8225
6000	41.1560(−0.001)	41.1562	41.965(−0.133)	41.9900(−0.073)	42.0206
7000	40.7789(−0.009)	40.7825		42.2439(−0.123)	42.2961
8000	39.5528(−0.001)	39.5532		41.5222(−0.162)	41.5896
9000	37.8356(0.022)	37.8273		40.0827(−0.183)	40.1563
10000	35.9794(0.128)	35.9333		38.2676(−0.189)	38.3400

a Relative percentage errors with respect to present values are given in parentheses.

b Reference (31).

c Reference (27).

d Reference (26).

As observed, there is a fairly good agreement between the heat capacities obtained using these four methods up to 2000–3000 K, with relative errors that practically do not exceed 0.005% in absolute value. For higher temperatures, deviations from our results start to increase. The comparison is, logically, more limited for the heat capacities of the JANAF database, since they are given only up to 6000 K. These heat capacities are calculated using relatively old spectroscopic data,^27^ so they are expectedly to become more erroneous above 6000 K. As for the heat capacities determined by Popovas and Jorgensen, and by Le Roy, although the differences also increase with temperature, especially those by Le Roy, their values continue to show a good agreement with ours, with relative errors remaining below 0.2% in the whole range of temperatures considered. On the other hand, it is worth highlighting again the pronounced effect that inclusion of quasi-bound energy levels has on the heat capacity at high temperatures, for which, as seeing in Table 11, an absolute difference between our values using only bound levels and those including the quasi-bound levels of 2.4067 J^–1^ mol^–1^ is observed at the highest temperature of 10000 K, representing a relative error of −6.67%.

For the remaining thermodynamic quantities, the entropy *S*(*T*), the relative enthalpy *H*(*T*) – *H*_0_, and the reduced Gibbs free energy −(*G*(*T*) – *H*_0_)/*T*, which are less sensitive to the partition function, the agreement between our results for H_2_ and those by Popovas and Jorgenson, JANAF, and Le Roy et al. is quite good, as shown in the corresponding tables included in the Supporting Information.

For the rest of isotopologues, we have for comparison the JANAF data for HD and D_2_, and the Le Roy et al. results for all of them, HD, D_2_, HT, DT, and T_2_. In Table 12 we include the values of the heat capacities for the isotopologues HD and D_2_. As observed, the heat capacities determined by Le Roy et al. for these two isotopologues show deviations from our values similar to those for the main isotopologue H_2_, while the heat capacities provided by the JANAF database deteriorate more rapidly than those of H_2_ as the temperature rises, especially for the HD isotopologue.

**Table 12:** Values of *C*_*P*_^°^ (J K^–1^ mol^–1^) for the Isotopologues HD and D_2_ Calculated Using Different Methods Including Quasi-Bound Levels^a^

	HD			D_2_		
*T* (K)	JANAF^b^	Le Roy^c^	present-cbl	JANAF	Le Roy	present-cbl
10		20.7972(0.001)	20.7971		20.9559(0.001)	20.9557
50		29.9130(0.001)	29.9128		29.0290(0.001)	29.0288
100	29.288(0.007)	29.2866(0.001)	29.2864	30.317(−0.003)	30.3185(0.000)	30.3184
150		29.1963(0.001)	29.1961		29.4158(0.001)	29.4156
200	29.188(0.007)	29.1861(0.001)	29.1859	29.204(−0.003)	29.2056(0.001)	29.2054
250		29.1911(0.001)	29.1909	29.185(−0.003)	29.1859(0.000)	29.1858
300	29.202(0.003)	29.2012(0.001)	29.2010	29.195(−0.004)	29.1962(0.000)	29.1961
400	29.231(0.003)	29.2299(0.001)	29.2297	29.242(−0.007)	29.2440(0.001)	29.2438
500	29.283(0.003)	29.2826(0.001)	29.2824	29.366(−0.007)	29.3686(0.001)	29.3683
600	29.395(0.007)	29.3935(0.001)	29.3932	29.619(−0.010)	29.6221(0.001)	29.6217
700	29.594(0.007)	29.5928(0.002)	29.5923	30.008(−0.010)	30.0116(0.002)	30.0111
800	29.890(0.010)	29.8881(0.002)	29.8874	30.502(−0.010)	30.5060(0.002)	30.5054
900	30.269(0.010)	30.2668(0.003)	30.2659	31.057(−0.016)	31.0622(0.002)	31.0615
1000	30.708(0.010)	30.7058(0.003)	30.7048	31.636(−0.016)	31.6417(0.003)	31.6409
2000	35.050(0.029)	35.0414(0.004)	35.0400	35.975(−0.033)	35.9877(0.002)	35.9869
3000	37.625(0.080)	37.5958(0.003)	37.5946	38.156(−0.039)	38.1715(0.002)	38.1709
4000	39.513(0.198)	39.4357(0.002)	39.4348	39.785(−0.032)	39.7981(0.000)	39.7981
5000	41.185(0.329)	41.0486(−0.004)	41.0501	41.262(−0.073)	41.2884(−0.009)	41.2922
6000	42.339(0.398)	42.1623(−0.020)	42.1707	42.353(−0.196)	42.3233(−0.030)	42.3363
7000		42.3703(−0.044)	42.3891		42.4761(−0.060)	42.5017
8000		41.6122(−0.071)	41.6416		41.6789(−0.089)	41.7160
9000		40.1435(−0.093)	40.1809		40.1835(−0.111)	40.2283
10000		38.3057(−0.110)	38.3479		38.3283(−0.126)	38.3766

a Relative percentage errors with respect to present values are given in parentheses.

b Reference (27).

c Reference (26).

**Figure 7:**
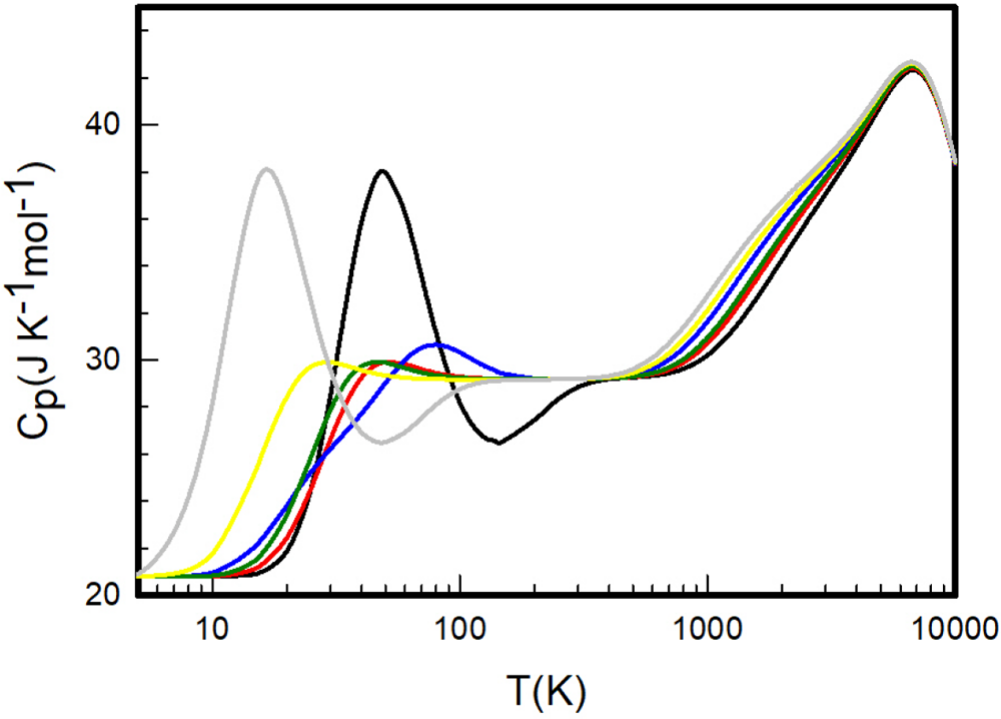
Heat capacities at constant pressure *C*_*P*_ for the molecular hydrogen isotopologues H_2_ (black), HD (red), D_2_ (blue), HT (dark green), DT (yellow), and T_2_ (gray), calculated in this work.

In Table 13, we include the heat capacities *C*_*P*_ for the six isotopologues of molecular hydrogen obtained in this work using the Pachucki and Komasa adiabatic potentials, which are also plotted together in Figure 7 versus temperature to better visualize them.

**Table 13:** Values of *C*_*P*_^°^ (J K^–1^ mol^–1^) for the H_2_ Isotopologues Calculated Using the Rovibrational Energy Levels Extracted from the Adiabatic Pachoucki and Komasa Potentials^34,35^ Including the Quasi-Bound Levels

*T* (K)	H_2_	HD	D_2_	HT	DT	T_2_
10	20.7870	20.7971	20.9557	20.8215	21.7578	28.1656
50	37.9703	29.9128	29.0288	29.8862	29.3383	26.5040
100	28.1535	29.2864	30.3184	29.2404	29.1632	28.7619
150	26.5559	29.1961	29.4156	29.1835	29.1582	29.1346
200	27.4475	29.1859	29.2054	29.1799	29.1671	29.1637
250	28.3446	29.1909	29.1858	29.1873	29.1797	29.1788
300	28.8490	29.2010	29.1961	29.1987	29.1959	29.2005
400	29.1814	29.2297	29.2438	29.2309	29.2651	29.3160
500	29.2600	29.2824	29.3683	29.2971	29.4513	29.6098
600	29.3268	29.3932	29.6217	29.4394	29.8004	30.0982
700	29.4404	29.5923	30.0111	29.6858	30.2937	30.7214
800	29.6232	29.8874	30.5054	30.0355	30.8785	31.4044
900	29.8803	30.2659	31.0615	30.4669	31.5019	32.0897
1000	30.2039	30.7048	31.6409	30.9511	32.1243	32.7427
2000	34.2769	35.0400	35.9869	35.3320	36.3526	36.7480
3000	37.0778	37.5946	38.1709	37.7793	38.3773	38.5918
4000	39.0955	39.4348	39.7981	39.5538	39.9250	40.0550
5000	40.8225	41.0501	41.2922	41.1353	41.3788	41.4675
6000	42.0206	42.1707	42.3363	42.2421	42.4036	42.4719
7000	42.2961	42.3891	42.5017	42.4569	42.5601	42.6171
8000	41.5896	41.6416	41.7160	41.7081	41.7697	41.8181
9000	40.1563	40.1809	40.2283	40.2447	40.2779	40.3182
10000	38.3400	38.3479	38.3766	38.4069	38.4219	38.4542

Let us finally consider the thermodynamic quantities of the normal mixtures of the homonuclear isotopologues H_2_, D_2_, and T_2_. As already indicated in Section 2, the Colonna et al. treatment^30^ of normal mixtures ensures that the thermodynamic quantities directly depending on the partition function match the equilibrium values in the high-temperature limit. For practical purposes, such a limit is reached at relatively low temperatures for the three isotopologues, not higher than 300 K, in which range our values for the thermodynamic quantities of the normal mixtures agree well with those obtained by Popovas and Jorgensen^31^ and by Colonna et al.^30^ for the main isotopologue H_2_, and with those by Le Roy et al.^26^ for the three homonuclear isotopologues H_2_, D_2_, and T_2_.

**Figure 8:**
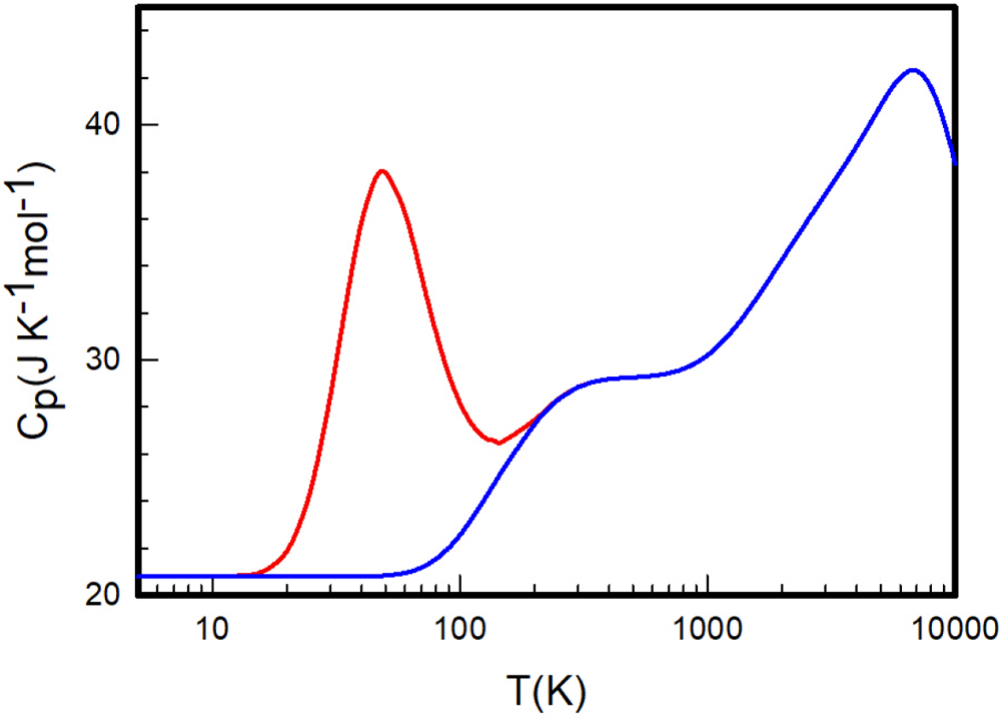
Heat capacities at constant pressure *C*_*P*_ of equilibrium (red) and normal (blue) H_2_ calculated by summation over the *ab initio* rovibrational energy levels determined by Pachucki and Komasa.

**Figure 9:**
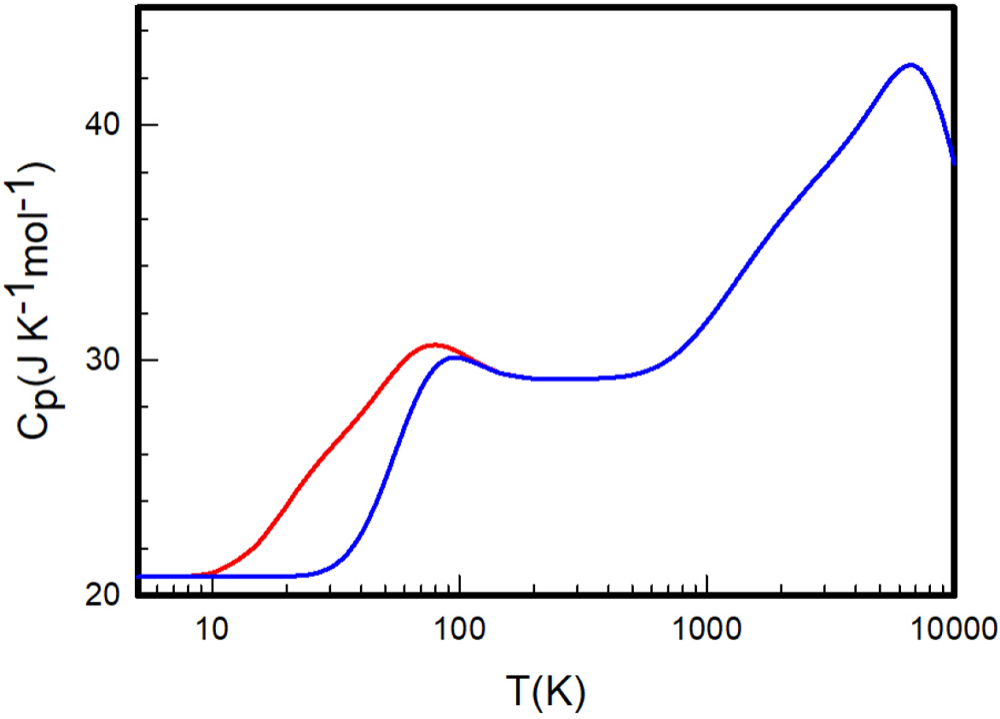
Heat capacities at constant pressure *C*_*P*_ of equilibrium (red) and normal (blue) D_2_ calculated by summation over the *ab initio* rovibrational energy levels determined by Pachucki and Komasa.

**Figure 10:**
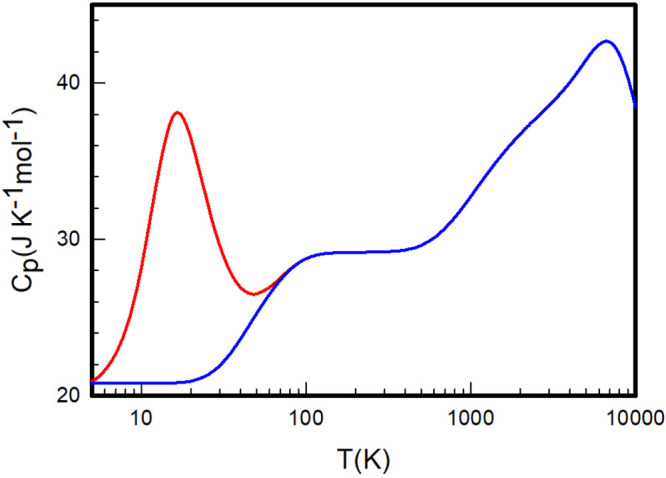
Heat capacities at constant pressure *C*_*P*_ of equilibrium (red) and normal (blue) T_2_ calculated by summation over the *ab initio* rovibrational energy levels determined by Pachucki and Komasa.

In Table 14, we give the values of the four thermodynamic quantities *C*_*P*_(*T*), *S*(*T*), *H*(*T*) – *H*_0_, and −(*G*(*T*) – *H*_0_)/*T* for the normal and equilibrium mixtures of the main isotopologue H_2_. In this table, we include a greater number of temperatures below 300 K, where the differences between the normal and equilibrium mixtures are more pronounced, and limit the highest temperature to 600 K, at which the thermodynamic quantities of both mixtures already coincide. The differences between the four thermodynamic quantities of the normal and equilibrium mixtures of H_2_ are clearly noticeable at the temperatures below 300 K, as they are for the normal mixtures of the other two homonuclear isotopologues D_2_ and T_2_, which are shown in the tables containing their thermodynamic quantities included in the Supporting Information, and in Figures 8, 9, and 10 for the heat capacities *C*_*P*_ of the three homonuclear isotopologues.

**Table 14:** Thermodynamic Quantities of the Main Isotopologue H_2_ for Equilibrium Mixtures and Normal Mixtures Calculated Using the Method of Colonna et al.,^30^ Both Obtained Using the Rovibrational Energy Levels Extracted from the Adiabatic Pachoucki and Komasa Potentials^34,35^ and Including Quasi-Bound Levels

	*C*_*P*_^°^ (J K^–1^ mol^–1^)		*S*° (J K^–1^ mol^–1^)		*H*° – *H*°(0) (kJ mol^–1^)		–(*G*° – *H*°(0))/*T* (J K^–1^ mol^–1^)	
*T* (K)	normal	equilibrium	normal	equilibrium	normal	equilibrium	normal	equilibrium
5	20.7862	20.7862	50.9972	32.6201	1.16704	0.103931	–182.411	11.8340
10	20.7862	20.7870	65.4051	47.0280	1.27097	0.207862	–61.6920	26.2418
20	20.7862	21.8622	79.8129	61.5772	1.47883	0.418252	5.87133	40.6646
30	20.7863	28.5310	88.2410	71.5194	1.68669	0.665722	32.0179	49.3287
40	20.7911	35.8890	94.2212	80.8268	1.89457	0.991049	46.8570	56.0505
50	20.8266	37.9703	98.8634	89.1594	2.10262	1.36485	56.8112	61.8623
60	20.9411	36.2456	102.669	95.9625	2.31137	1.73773	64.1464	67.0004
70	21.1742	33.5409	105.913	101.348	2.52184	2.08671	69.8868	71.5374
80	21.5365	31.1567	108.763	105.664	2.73529	2.40972	74.5717	75.5427
90	22.0102	29.3802	111.326	109.226	2.95294	2.71190	78.5154	79.0932
100	22.5620	28.1535	113.673	112.253	3.17575	2.99916	81.9153	82.2616
150	25.3835	26.5559	123.373	123.191	4.37675	4.34543	94.1946	94.2219
200	27.2684	27.4475	130.957	130.938	5.69721	5.69291	102.471	102.473
250	28.3225	28.3446	137.168	137.166	7.08978	7.08928	108.809	108.809
300	28.8465	28.8490	142.384	142.384	8.52066	8.52060	113.982	113.982
400	29.1813	29.1814	150.742	150.742	11.4265	11.4265	122.175	122.175
500	29.2600	29.2600	157.263	157.263	14.3492	14.3492	128.565	128.565
600	29.3268	29.3268	162.603	162.603	17.2783	17.2783	133.806	133.806

Finally, for completeness, in Table 15 we give the enthalpies at the reference temperature and the values of the thermodynamic quantities of the molecular hydrogen isotopologues at the standard temperature of 298.15 K.

**Table 15:** Reference Zero-Point Enthalpies and Thermodynamic Magnitudes at 298.15 K for Molecular Hydrogen Isotopologues

	*H*°(0)^a^	*C*_*P*_^°^	*S*°	*H*° – *H*°(0)	*G*° – *H*°(0)	–(*G*° – *H*°(0))/*T*
H_2_	–432.072	28.8362	142.205	8.46724	–33.9313	113.806
H_2_(*n*)	–431.008	28.8336	142.206	8.46730	–33.9313	113.806
HD	–435.515	29.2006	158.700	8.50874	–38.8076	130.161
HT	–436.788	29.1982	159.867	8.52885	–39.1354	131.261
D_2_	–439.615	29.1955	163.230	8.56910	–40.0978	134.489
D_2_(*n*)	–439.377	29.1955	163.230	8.56910	–40.0978	134.489
DT	–441.205	29.1952	169.890	8.58917	–42.0636	141.082
T_2_	–442.967	29.1995	164.855	8.60930	–40.5422	135.979
T_2_(*n*)	–442.607	29.1995	164.855	8.60930	–40.5422	135.979

a *H*°(0) = *E*°(0) = *G*°(0).

## 4 Conclusions

In this work, we have calculated the partition functions and thermodynamic quantities of the six isotopologues of molecular hydrogen, H_2_, HD, HT, D_2_, DT, and T_2_, using the ro-vibrational energy levels extracted from the adiabatic potentials recently determined by Pachucki and Komasa employing high-level *ab initio* methods.^34^ For this purpose, we have recalculated the bound energy levels of the adiabatic potentials using the HEG linear variation method and estimated the quasi-bound energy levels lying below the centrifugal potential barriers by leveling the barriers beyond their maxima. We have also determined the partition functions and thermodynamic quantities of the normal mixtures of the homonuclear isotopologues H_2_, D_2_, and T_2_ using the thermodynamic-statistical method of Colonna et al.,^30^ which eliminates inconsistencies in their values with respect to equilibrium mixtures in the high-temperature limit.

The excellent agreement between the partition functions of the main isotopologue H_2_ obtained using the rovibrational energy levels of the *ab inito* adiabatic potentials and those calculated by Popovas and Jorgensen^31^ using a highly accurate empirical Dunham expansion, up to 10000 K, confirms the accuracy of the partition functions *ab initio* calculated in this work for the H_2_ isotopologue, which are certainly to be shared by the partition functions determined for the remaining isotopologues. Such agreement also extends to the partition functions calculated for the normal mixtures of the homonuclear isotopologues H_2_, D_2_, and T_2_. In addition, the inclusion of quasi-bound energy levels in the calculation of the partition functions of molecular hydrogen isotopologues is shown to become increasingly important, and therefore necessary, for temperatures higher than 5000–6000 K.

The above conclusions are further supported by the excellent agreement between the *ab initio* thermodynamic functions calculated using the Pachucky and Komasa adiabatic potentials and those obtained empirically by Popovas and Jorgensen for the main isotopologue H_2_, and theoretically by Le Roy et al.^26^ for the six isotopologues employing less elaborated *ab initio* potential functions.

In the Supporting Information, we give the partition functions and thermodynamic quantities of the six isotopologues of molecular hydrogen calculated in this work in the range of temperatures between 1 and 10000 K, in steps of 1 K, for them to be useful in the study of chemical-physical phenomena in which these isotopologues play an important and/or fundamental role.

## Supporting Information Available

The minima and maxima of the effective potentials of the hydrogen isotopologues, the quasi-bound energy levels of the hydrogen isotopologues below the centrifugal barrier maxima, the values of the thermodynamic quantities *S*°, *H*° – *H*°, and −(*G*° – *H*°(*T*_ref._))/*T* for the main isotopologue H_2_ calculated using different methods, and the thermodynamic quantities of the isotopologues D_2_ and T_2_ for equilibrium and normal mixtures (PDF)
The partition functions and thermodynamic quantities of the six isotopologues of molecular hydrogen in the range of temperatures between 1 and 10000 K, in steps of 1 K (ZIP)
