# Correction to “*Ab Initio* Partition Functions and Thermodynamic Quantities for the Molecular Hydrogen Isotopologues”

**DOI:** 10.1021/acs.jpca.4c04212

**Published:** 2024-07-08

**Authors:** José Zúñiga, Adolfo Bastida, Alberto Requena, Javier Cerezo

In our original manuscript, Equations 4, (6), (19), (20), (23), (24), (27), and (28) should read as follows
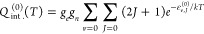
4
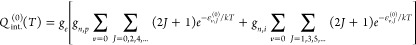
6
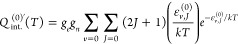
19
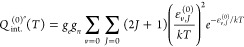
20
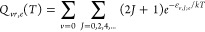
23
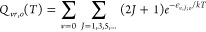
24
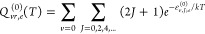
27
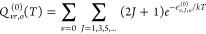
28

The corrections consist of replacing the *J*(*J* + 1) terms by the (2*J* + 1) terms in the above equations. The errors are typographical and do not affect any of the results nor the conclusions of our original work.

