# Supplementary material for: Ab Initio Partition Functions and Thermodynamic Quantities for the Molecular Hydrogen Isotopologues
Source: J Phys Chem A. 2021 Oct 6;125(41):9226–41. doi: 10.1021/acs.jpca.1c06468 (PMC8543445; doi:10.1021/acs.jpca.1c06468)
Supplement: Supplementary file 1 — jp1c06468_si_001.pdf [file jp1c06468_si_001.pdf]

Ab Initio Partition Functions and Thermodynamic Quantities for the Molecular Hydrogen Isotopologues (SUPPORTING INFORMATION)

José Zúñiga,*,† Adolfo Bastida,† Alberto Requena,† and Javier Cerezo*,‡

Departamento de Química Física, Universidad de Murcia, 30100 Murcia, Spain, and Departamento de Química, Universidad Autónoma de Madrid, 28049, Madrid, Spain

E-mail: zuniga@um.es; javier.cerezo@uam.es

*To whom correspondence should be addressed

†Departamento de Química Física, Universidad de Murcia, 30100 Murcia, Spain

‡Departamento de Química, Universidad Autónoma de Madrid, 28049, Madrid, Spain

Table S1: Minima and maxima of the effective potentials of the parent isotopologue H_2.

J	R_o(Å)	V_o(cm^{-1})	R_b(Å)	V_b(cm^{-1})
0	0.7416254	-38298.019151	0.000000	0.000000
1	0.7427571	-38176.491208	6.462004	1.102407
2	0.7450145	-37934.541314	5.272606	5.246364
3	0.7483856	-37574.346706	4.769537	13.35797
4	0.7528530	-37099.088930	4.461242	26.03086
5	0.7583952	-36512.859489	4.238008	43.80553
6	0.7649864	-35820.544475	4.061843	67.20030
7	0.7725986	-35027.695416	3.915500	96.72020
8	0.7812018	-34140.393673	3.789745	132.8630
9	0.7907653	-33165.115155	3.679058	176.1238
10	0.8012586	-32108.601036	3.579876	226.9986
11	0.8126522	-30977.738856	3.489759	285.9875
12	0.8249183	-29779.456982	3.406959	353.5974
13	0.8380317	-28520.634111	3.330182	430.3442
14	0.8519699	-27208.024355	3.258435	516.7560
15	0.8667136	-25848.197588	3.190941	613.3744
16	0.8822476	-24447.494132	3.127077	720.7582
17	0.8985608	-23011.992405	3.066332	839.4854
18	0.9156466	-21547.488017	3.008283	970.1563
19	0.9335033	-20059.482716	2.952569	1113.397
20	0.9521347	-18553.181653	2.898881	1269.863
21	0.9715505	-17033.497579	2.846946	1440.244
22	0.9917671	-15505.060778	2.796522	1625.268
23	1.012809	-13972.233749	2.747388	1825.708
24	1.034708	-12439.129894	2.699340	2042.389
25	1.057507	-10909.635729	2.652182	2276.197
26	1.081263	-9387.4364084	2.605724	2528.089
27	1.106046	-7876.0446849	2.559777	2799.109
28	1.131946	-6378.8338349	2.514142	3090.399
29	1.159077	-4899.0756198	2.468608	3403.230
30	1.187589	-3439.9851650	2.422940	3739.023
31	1.217674	-2004.7759105	2.376866	4099.394
32	1.249591	-596.72995129	2.330057	4486.209
33	1.283691	780.70697206	2.282094	4901.664
34	1.320476	2123.7888298	2.232415	5348.408
35	1.360698	3428.2573833	2.180216	5829.746
36	1.405562	4688.9813317	2.124239	6349.986
37	1.457235	5899.2599705	2.062269	6915.124
38	1.520409	7049.1996226	1.989574	7534.459
39	1.610548	8120.2806688	1.890646	8226.017

Table S2: Minima and maxima of the effective potential of the isotopologue HD.

J	R_o(Å)	V_o(cm^{-1})	R_b(Å)	V_b(cm^{-1})
0	0.7415744	-38296.774032	0.000000	0.000000
1	0.7424235	-38205.565730	6.878090	0.7250263
2	0.7441184	-38023.772912	5.526396	3.519463
3	0.7466522	-37752.628379	4.962762	9.109071
4	0.7500150	-37393.945235	4.629853	17.91785
5	0.7541942	-36950.075788	4.394555	30.31063
6	0.7591748	-36423.860211	4.211771	46.63352
7	0.7649397	-35818.567515	4.061593	67.22147
8	0.7714705	-35137.831525	3.933599	92.40232
9	0.7787479	-34385.584548	3.821674	122.4998
10	0.7867520	-33565.991180	3.721924	157.8357
11	0.7954627	-32683.384392	3.631716	198.7319
12	0.8048606	-31742.205581	3.549185	245.5118
13	0.8149269	-30746.949866	3.472959	298.5020
14	0.8256439	-29702.117434	3.401999	358.0330
15	0.8369954	-28612.171395	3.335496	424.4411
16	0.8489667	-27481.502230	3.272808	498.0692
17	0.8615452	-26314.398678	3.213416	579.2678
18	0.8747201	-25115.024730	3.156891	668.3968
19	0.8884830	-23887.402220	3.102876	765.8264
20	0.9028281	-22635.398487	3.051067	871.9383
21	0.9177521	-21362.718511	3.001204	987.1280
22	0.9332545	-20072.900941	2.953061	1111.806
23	0.9493381	-18769.317488	2.906438	1246.398
24	0.9660087	-17455.175167	2.861157	1391.351
25	0.9832762	-16133.520956	2.817057	1547.133
26	1.001154	-14807.248521	2.773991	1714.235
27	1.019661	-13479.106691	2.731821	1893.176
28	1.038821	-12151.709497	2.690416	2084.507
29	1.058664	-10827.547628	2.649652	2288.815
30	1.079226	-9509.0012802	2.609406	2506.727
31	1.100554	-8198.3544805	2.569553	2738.920
32	1.122704	-6897.8110816	2.529966	2986.126
33	1.145745	-5609.5128237	2.490512	3249.145
34	1.169765	-4335.5600829	2.451044	3528.857
35	1.194870	-3078.0362874	2.411402	3826.239
36	1.221198	-1839.0375330	2.371398	4142.386
37	1.248926	-620.70982413	2.330810	4478.544
38	1.278285	574.70209492	2.289364	4836.150
39	1.309589	1744.7875534	2.246706	5216.895
40	1.343281	2886.8722078	2.202358	5622.813

Table S2: Continued.

J	R_o(Å)	V_o(cm^{-1})	R_b(Å)	V_b(cm^{-1})
41	1.380011	3997.8660245	2.155635	6056.429
42	1.420806	5074.0062123	2.105478	6521.013
43	1.467464	6110.3705272	2.050062	7021.055
44	1.523695	7099.7856630	1.985646	7563.359
45	1.600364	8029.4952680	1.901341	8160.367

Table S3: Minima and maxima of the effective potential of the isotopologue HT.

J	R_o(Å)	V_o(cm^{-1})	R_b(Å)	V_b(cm^{-1})
0	0.7415574	-38296.360078	0.000000	0.000000
1	0.7423125	-38215.242929	7.061402	0.6103988
2	0.7438201	-38053.502236	5.640329	2.983947
3	0.7460746	-37812.114805	5.047256	7.776327
4	0.7490684	-37492.520341	4.702040	15.36211
5	0.7527912	-37096.592245	4.460656	26.05317
6	0.7572310	-36626.600880	4.274483	40.14346
7	0.7623740	-36085.170942	4.122283	57.91667
8	0.7682052	-35475.234725	3.993046	79.64980
9	0.7747087	-34799.983073	3.880359	105.6156
10	0.7818680	-34062.815729	3.780165	136.0846
11	0.7896662	-33267.292593	3.689737	171.3264
12	0.7980869	-32417.087176	3.607150	211.6113
13	0.8071137	-31515.943252	3.530993	257.2112
14	0.8167311	-30567.635425	3.460202	308.4007
15	0.8269245	-29575.934087	3.393952	365.4579
16	0.8376806	-28544.574979	3.331590	428.6653
17	0.8489871	-27477.233398	3.272588	498.3112
18	0.8608334	-26377.502906	3.216513	574.6896
19	0.8732105	-25248.878315	3.163005	658.1021
20	0.8861113	-24094.742615	3.111761	748.8583
21	0.8995305	-22918.357482	3.062520	847.2771
22	0.9134649	-21722.856984	3.015059	953.6874
23	0.9279135	-20511.244091	2.969180	1068.430
24	0.9428777	-19286.389626	2.924710	1191.858
25	0.9583615	-18051.033323	2.881494	1324.340
26	0.9743717	-16807.786675	2.839391	1466.259
27	0.9909181	-15559.137334	2.798273	1618.019
28	1.008014	-14307.454824	2.758019	1780.041
29	1.025677	-13054.997422	2.718518	1952.770
30	1.043928	-11803.920073	2.679661	2136.680
31	1.062794	-10556.283298	2.641346	2332.270
32	1.082307	-9314.0630850	2.603467	2540.078
33	1.102509	-8079.1618307	2.565922	2760.676
34	1.123446	-6853.4205169	2.528603	2994.686
35	1.145178	-5638.6323763	2.491397	3242.783
36	1.167778	-4436.5584963	2.454183	3505.704
37	1.191335	-3248.9460216	2.416825	3784.263
38	1.215961	-2077.5499666	2.379171	4079.367
39	1.241797	-924.16019523	2.341038	4392.038
40	1.269026	209.36396323	2.302209	4723.441

Table S3: Continued.

J	R_o(Å)	V_o(cm^{-1})	R_b(Å)	V_b(cm^{-1})
41	1.297888	1321.0474187	2.262409	5074.926
42	1.328713	2408.7343790	2.221278	5448.091
43	1.361963	3469.9920350	2.178324	5844.872
44	1.398332	4501.9577052	2.132827	6267.689
45	1.438929	5501.0742336	2.083650	6719.713
46	1.485749	6462.5733902	2.028775	7205.372
47	1.543120	7379.2600660	1.963851	7731.570
48	1.625476	8237.3928069	1.874424	8311.799

Table S4: Minima and maxima of the effective potential of the isotopologue D_2.

J	R_o(Å)	V_o(cm^{-1})	R_b(Å)	V_b(cm^{-1})
0	0.7415233	-38295.529669	0.000000	0.000000
1	0.7420898	-38234.672457	7.542904	0.3996325
2	0.7432211	-38113.236107	5.948475	1.982349
3	0.7449142	-37931.772354	5.271367	5.252177
4	0.7471648	-37691.097940	4.889236	10.49557
5	0.7499669	-37392.282028	4.629407	17.92828
6	0.7533135	-37036.630144	4.432865	27.74830
7	0.7571967	-36625.665203	4.274372	40.14521
8	0.7616073	-36161.106233	4.141142	55.30305
9	0.7665357	-35644.845470	4.025873	73.40203
10	0.7719716	-35078.924480	3.924024	94.61982
11	0.7779044	-34465.509925	3.832579	119.1325
12	0.7843232	-33806.869563	3.749437	147.1153
13	0.7912171	-33105.348969	3.673074	178.7436
14	0.7985755	-32363.349398	3.602348	214.1931
15	0.8063879	-31583.307121	3.536381	253.6404
16	0.8146440	-30767.674457	3.474485	297.2640
17	0.8233344	-29918.902681	3.416105	345.2442
18	0.8324501	-29039.426865	3.360793	397.7636
19	0.8419829	-28131.652695	3.308177	455.0078
20	0.8519252	-27197.945208	3.257943	517.1656
21	0.8622706	-26240.619399	3.209830	584.4297
22	0.8730134	-25261.932566	3.163611	656.9967
23	0.8841490	-24264.078297	3.119090	735.0681
24	0.8956739	-23249.181940	3.076098	818.8504
25	0.9075857	-22219.297426	3.034484	908.5558
26	0.9198833	-21176.405298	2.994115	1004.403
27	0.9325668	-20122.411818	2.954872	1106.617
28	0.9456378	-19059.149008	2.916646	1215.432
29	0.9590992	-17988.375525	2.879340	1331.088
30	0.9729557	-16911.778255	2.842864	1453.836
31	0.9872136	-15830.974538	2.807133	1583.938
32	1.001881	-14747.514946	2.772069	1721.666
33	1.016969	-13662.886555	2.737599	1867.304
34	1.032490	-12578.516664	2.703650	2021.152
35	1.048459	-11495.776935	2.670155	2183.524
36	1.064895	-10415.987942	2.637046	2354.753
37	1.081820	-9340.4241432	2.604257	2535.190
38	1.099260	-8270.3193100	2.571718	2725.210
39	1.117245	-7206.8724815	2.539362	2925.214
40	1.135813	-6151.2545414	2.507116	3135.630

Table S4: Continued.

J	R_o(Å)	V_o(cm^{-1})	R_b(Å)	V_b(cm^{-1})
41	1.155008	-5104.6155746	2.474903	3356.923
42	1.174880	-4068.0932184	2.442640	3589.595
43	1.195495	-3042.8223246	2.410235	3834.197
44	1.216927	-2029.9463837	2.377586	4091.333
45	1.239272	-1030.6313745	2.344572	4361.677
46	1.262646	-46.083034084	2.311053	4645.980
47	1.287200	922.43092480	2.276858	4945.099
48	1.313125	1873.5512057	2.241772	5260.017
49	1.340679	2805.7858606	2.205519	5591.887
50	1.370217	3717.4505996	2.167727	5942.086
51	1.402249	4606.5769930	2.127867	6312.306
52	1.437554	5470.7608294	2.085142	6704.705
53	1.477438	6306.8863774	2.038234	7122.181
54	1.524394	7110.5494864	1.984632	7568.944
55	1.584575	7874.5297177	1.918170	8052.046
56	1.691773	8581.5521014	1.805043	8588.617

Table S5: Minima and maxima of the effective potential of the isotopologue DT.

J	R_o(Å)	V_o(cm^{-1})	R_b(Å)	V_b(cm^{-1})
0	0.7415064	-38295.115966	0.000000	0.000000
1	0.7419787	-38244.360379	7.865260	0.3059160
2	0.7429224	-38143.042709	6.166181	1.526588
3	0.7443352	-37991.547405	5.427813	4.084336
4	0.7462141	-37790.444807	5.016652	8.225489
5	0.7485549	-37540.483778	4.742064	14.12418
6	0.7513528	-37242.582222	4.537159	21.93554
7	0.7546019	-36897.815782	4.373530	31.80698
8	0.7582958	-36507.405024	4.236972	43.88107
9	0.7624275	-36072.701461	4.119477	58.29696
10	0.7669895	-35595.172762	4.016116	75.19142
11	0.7719739	-35076.387491	3.923650	94.69953
12	0.7773726	-34517.999712	3.839836	116.9553
13	0.7831775	-33921.733748	3.763059	142.0924
14	0.7893801	-33289.369370	3.692117	170.2440
15	0.7959722	-32622.727627	3.626091	201.5440
16	0.8029459	-31923.657501	3.564261	236.1266
17	0.8102933	-31194.023520	3.506054	274.1272
18	0.8180068	-30435.694433	3.451004	315.6822
19	0.8260795	-29650.532996	3.398728	360.9297
20	0.8345046	-28840.386890	3.348906	410.0097
21	0.8432761	-28007.080789	3.301269	463.0639
22	0.8523884	-27152.409524	3.255587	520.2367
23	0.8618365	-26278.132320	3.211663	581.6749
24	0.8716162	-25385.968040	3.169323	647.5285
25	0.8817238	-24477.591367	3.128419	717.9504
26	0.8921567	-23554.629860	3.088818	793.0972
27	0.9029126	-22618.661793	3.050402	873.1294
28	0.9139905	-21671.214724	3.013065	958.2117
29	0.9253900	-20713.764701	2.976712	1048.513
30	0.9371118	-19747.736043	2.941256	1144.209
31	0.9491573	-18774.501638	2.906620	1245.478
32	0.9615294	-17795.383682	2.872729	1352.507
33	0.9742318	-16811.654828	2.839517	1465.489
34	0.9872695	-15824.539680	2.806921	1584.622
35	1.000649	-14835.216599	2.774880	1710.116
36	1.014378	-13844.819804	2.743339	1842.186
37	1.028466	-12854.441716	2.712243	1981.059
38	1.042925	-11865.135558	2.681539	2126.971
39	1.057767	-10877.918189	2.651176	2280.172
40	1.073008	-9893.7731762	2.621103	2440.924

Table S5: Continued.

J	R_o(Å)	V_o(cm^{-1})	R_b(Å)	V_b(cm^{-1})
41	1.088668	-8913.6541304	2.591269	2609.503
42	1.104766	-7938.4883174	2.561622	2786.203
43	1.121328	-6969.1806037	2.532108	2971.336
44	1.138384	-6006.6177923	2.502671	3165.236
45	1.155968	-5051.6734427	2.473253	3368.261
46	1.174119	-4105.2133015	2.443790	3580.796
47	1.192887	-3168.1015205	2.414212	3803.262
48	1.212329	-2241.2079078	2.384440	4036.115
49	1.232513	-1325.4165618	2.354387	4279.857
50	1.253524	-421.63639291	2.323950	4535.045
51	1.275466	469.18572409	2.293008	4802.302
52	1.298471	1346.0480359	2.261412	5082.332
53	1.322707	2207.8714602	2.228980	5375.941
54	1.348394	3053.4686932	2.195476	5684.068
55	1.375833	3881.4998746	2.160587	6007.826
56	1.405446	4690.4056559	2.123875	6348.571
57	1.437865	5478.2995207	2.084698	6708.003
58	1.474102	6242.7793706	2.042031	7088.360
59	1.515984	6980.5561569	1.994037	7492.783
60	1.567500	7686.5702239	1.936714	7926.200
61	1.642291	8350.9325848	1.856413	8398.387

Table S6: Minima and maxima of the effective potential of the isotopologue T_2.

J	R_o(Å)	V_o(cm^{-1})	R_b(Å)	V_b(cm^{-1})
0	0.7414894	-38294.702346	0.000000	0.000000
1	0.7418677	-38254.051871	8.287833	0.2201958
2	0.7426236	-38172.875098	6.459122	1.105773
3	0.7437557	-38051.419065	5.638037	2.991538
4	0.7452620	-37890.051063	5.184603	6.083281
5	0.7471399	-37689.254809	4.887947	10.51924
6	0.7493861	-37449.625491	4.670441	16.41569
7	0.7519970	-37171.863805	4.499022	23.88145
8	0.7549681	-36856.769118	4.357364	33.02116
9	0.7582948	-36505.231910	4.236399	43.93655
10	0.7619721	-36118.225661	4.130619	56.72728
11	0.7659947	-35696.798332	4.036448	71.49142
12	0.7703568	-35242.063613	3.951435	88.32594
13	0.7750528	-34755.192082	3.873831	107.3271
14	0.7800767	-34237.402412	3.802343	128.5905
15	0.7854226	-33689.952763	3.735989	152.2120
16	0.7910846	-33114.132457	3.674007	178.2871
17	0.7970568	-32511.254032	3.615789	206.9118
18	0.8033336	-31882.645748	3.560847	238.1824
19	0.8099092	-31229.644608	3.508779	272.1960
20	0.8167784	-30553.589919	3.459255	309.0504
21	0.8239359	-29855.817433	3.411994	348.8445
22	0.8313771	-29137.654073	3.366759	391.6781
23	0.8390974	-28400.413243	3.323346	437.6524
24	0.8470925	-27645.390709	3.281582	486.8702
25	0.8553589	-26873.861044	3.241312	539.4356
26	0.8638930	-26087.074598	3.202402	595.4548
27	0.8726919	-25286.254981	3.164734	655.0356
28	0.8817533	-24472.597004	3.128203	718.2881
29	0.8910750	-23647.265073	3.092714	785.3247
30	0.9006556	-22811.391973	3.058183	856.2602
31	0.9104942	-21966.078029	3.024532	931.2122
32	0.9205903	-21112.390598	2.991692	1010.301
33	0.9309441	-20251.363865	2.959598	1093.650
34	0.9415565	-19383.998909	2.928192	1181.387
35	0.9524289	-18511.264013	2.897420	1273.643
36	0.9635635	-17634.095197	2.867229	1370.551
37	0.9749632	-16753.396940	2.837573	1472.251
38	0.9866317	-15870.043079	2.808407	1578.888
39	0.9985736	-14984.877870	2.779689	1690.609
40	1.010794	-14098.717189	2.751376	1807.569

Table S6: Continued.

J	R_o(Å)	V_o(cm^{-1})	R_b(Å)	V_b(cm^{-1})
41	1.023301	-13212.349866	2.723432	1929.930
42	1.036100	-12326.539149	2.695816	2057.858
43	1.049201	-11442.024289	2.668492	2191.528
44	1.062615	-10559.522242	2.641424	2331.123
45	1.076353	-9679.7295072	2.614575	2476.834
46	1.090429	-8803.3240930	2.587908	2628.862
47	1.104859	-7930.9676416	2.561385	2787.421
48	1.119661	-7063.3077239	2.534969	2952.735
49	1.134855	-6200.9803405	2.508620	3125.042
50	1.150466	-5344.6126725	2.482295	3304.595
51	1.166521	-4494.8261352	2.455950	3491.667
52	1.183053	-3652.2398156	2.429537	3686.548
53	1.200099	-2817.4743931	2.403002	3889.553
54	1.217705	-1991.1566871	2.376287	4101.022
55	1.235921	-1173.9250216	2.349325	4321.326
56	1.254813	-366.43567957	2.322042	4550.876
57	1.274456	430.62916832	2.294346	4790.121
58	1.294945	1216.5514960	2.266134	5039.568
59	1.316396	1990.5645665	2.237277	5299.785
60	1.338959	2751.8358718	2.207614	5571.424
61	1.362828	3499.4437367	2.176942	5855.240
62	1.388264	4232.3439792	2.144991	6152.123
63	1.415630	4949.3201438	2.111387	6463.150
64	1.445458	5648.9047676	2.075591	6789.662
65	1.478580	6329.2449575	2.036761	7133.396
66	1.516440	6987.8471719	1.993446	7496.743
67	1.561984	7621.0072095	1.942692	7883.312
68	1.623615	8222.0987016	1.876089	8299.650

Table S7: Quasi-bound energy levels (cm^{-1}) of the parent isotopologue H_2 below the centrifugal barrier maxima V_b.

J	v	Energy	$V_b(cm^{-1})$	J	v	Energy	$V_b(cm^{-1})$
4	14	0.983	26.03086	32	0	431.818	4486.209
5	14	43.263	43.80553	32	1	2270.389	4486.209
8	13	87.443	132.8630	32	2	3750.255	4486.209
11	12	212.899	285.9875	33	0	1743.596	4901.664
13	11	195.088	430.3442	33	1	3432.621	4901.664
14	11	477.406	516.7560	33	2	4687.129	4901.664
15	10	189.453	613.3744	34	0	3015.952	5348.408
16	10	583.296	720.7582	34	1	4534.487	5348.408
17	9	228.257	839.4854	35	0	4242.453	5829.746
18	9	722.239	970.1563	35	1	5548.925	5829.746
19	8	330.959	1113.397	36	0	5413.705	6349.986
19	9	1104.453	1113.397	37	0	6512.158	6915.124
20	8	917.207	1269.863	38	0	7486.563	7534.459
21	7	509.598	1440.244				
21	8	1399.876	1440.244				
22	7	1181.489	1625.268				
23	6	771.542	1825.708				
23	7	1751.371	1825.708				
24	5	233.159	2042.389				
24	6	1523.332	2042.389				
25	5	1121.167	2276.197				
25	6	2172.315	2276.197				
26	4	599.792	2528.089				
26	5	1948.019	2528.089				
27	4	1560.977	2799.109				
27	5	2670.793	2799.109				
28	3	1061.713	3090.399				
28	4	2459.087	3090.399				
29	2	473.385	3403.230				
29	3	2092.351	3403.230				
29	4	3252.733	3403.230				
30	2	1618.756	3739.023				
30	3	3059.118	3739.023				
31	1	1060.476	4099.394				
31	2	2716.064	4099.394				
31	3	3923.232	4099.394				

Table S8: Quasi-bound energy levels (cm^{-1}) of the isotopologue HD below the centrifugal barrier maxima V_b.

J	v	Energy	$V_b(cm^{-1})$	J	v	Energy	$V_b(cm^{-1})$
6	16	24.321	46.634	34	2	319.511	3528.857
9	15	25.380	122.500	34	3	1793.574	3528.857
10	15	138.884	157.836	34	4	2971.614	3528.857
12	14	99.396	245.512	35	2	1343.437	3826.239
13	14	270.894	298.502	35	3	2695.268	3826.239
14	13	29.414	358.033	35	4	3683.790	3826.239
15	13	290.254	424.441	36	1	834.642	4142.386
16	13	491.788	498.069	36	2	2334.574	4142.386
17	12	308.694	579.268	36	3	3544.175	4142.386
18	12	603.356	668.397	37	0	275.384	4478.544
19	11	352.156	765.826	37	1	1906.584	4478.544
20	11	726.213	871.938	37	2	3285.862	4478.544
21	10	436.196	987.128	37	3	4310.567	4478.544
22	9	10.764	1111.806	38	0	1422.162	4836.150
22	10	882.679	1111.806	38	1	2945.775	4836.150
23	9	570.951	1246.398	38	2	4185.929	4836.150
23	10	1234.824	1246.398	39	0	2540.600	5216.895
24	8	138.308	1391.351	39	1	3946.174	5216.895
24	9	1084.695	1391.351	39	2	5009.266	5216.895
25	8	763.119	1547.133	40	0	3627.033	5622.813
25	9	1510.332	1547.133	40	1	4898.348	5622.813
26	7	331.288	1714.235	41	0	4676.731	6056.429
26	8	1339.801	1714.235	41	1	5782.652	6056.429
27	7	1017.102	1893.176	42	0	5682.796	6521.013
27	8	1830.227	1893.176	42	1	6519.015	6521.013
28	6	592.220	2084.507	43	0	6632.702	7021.055
28	7	1653.025	2084.507	44	0	7492.641	7563.359
29	5	85.778	2288.815				
29	6	1335.762	2288.815				
29	7	2202.770	2288.815				
30	5	922.522	2506.727				
30	6	2027.830	2506.727				
31	4	432.468	2738.920				
31	5	1720.941	2738.930				
31	6	2633.279	2738.920				
32	4	1322.890	2986.126				
32	5	2466.714	2986.126				
33	3	851.851	3249.145				
33	4	2173.837	3249.145				
33	5	3125.790	3249.145				

Table S9: Quasi-bound energy levels (cm^{-1}) of the isotopologue HT below the centrifugal barrier maxima V_b.

J	v	Energy	$V_b(cm^{-1})$	J	v	Energy	$V_b(cm^{-1})$
2	18	2.876	2.984	34	4	1008.808	2994.686
6	17	4.098	40.143	34	5	2173.993	2994.686
7	17	53.219	57.917	34	6	2960.398	2994.686
10	16	91.552	136.085	35	3	532.894	3242.783
12	15	17.126	211.611	35	4	1843.952	3242.783
13	15	187.416	257.211	35	5	2868.324	3242.783
14	15	308.388	308.401	36	2	5.423	3505.704
15	14	160.896	365.458	36	3	1444.942	3505.704
16	14	375.081	428.665	36	4	2639.272	3505.704
17	13	133.725	498.311	36	5	3461.366	3505.704
18	13	423.727	574.690	37	2	988.411	3784.263
19	12	127.458	658.102	37	3	2325.030	3784.263
19	13	649.988	658.102	37	4	3379.739	3784.263
20	12	487.137	748.858	38	1	482.491	4079.367
21	11	156.153	847.277	38	2	1944.283	4079.367
21	12	793.116	847.277	38	3	3165.284	4079.367
22	11	581.213	953.687	38	4	4022.089	4079.367
23	10	229.316	1068.430	39	1	1508.139	4392.038
23	11	955.446	1068.430	39	2	2868.100	4392.038
24	10	716.008	1191.858	39	3	3951.813	4392.038
25	9	353.372	1324.340	40	0	1024.453	4723.441
25	10	1152.267	1324.340	40	1	2506.501	4723.441
26	9	898.252	1466.259	40	2	3752.803	4723.441
27	8	532.615	1618.019	40	3	4646.101	4723.441
27	9	1392.028	1618.019	41	0	2091.326	5074.926
28	7	81.029	1780.041	41	1	3473.301	5074.926
28	8	1132.515	1780.041	41	2	4586.178	5074.926
28	9	1775.494	1780.041	42	0	3131.241	5448.091
29	7	769.848	1952.770	42	1	4402.567	5448.091
29	8	1680.195	1952.770	42	2	5336.527	5448.091
30	6	326.950	2136.680	43	0	4140.753	5844.872
30	7	1421.909	2136.680	43	1	5284.392	5844.872
30	8	2122.262	2136.680	44	0	5115.277	6267.689
31	6	1066.843	2332.270	44	1	6095.678	6267.689
31	7	2020.555	2332.270	45	0	6047.817	6719.713
32	5	636.123	2540.078	46	0	6924.669	7205.373
32	6	1768.571	2540.078	47	0	7704.247	7731.570
32	7	2515.427	2540.078				
33	4	142.307	2760.676				
33	5	1424.671	2760.676				
33	6	2415.876	2760.676				

Table S10: Quasi-bound energy levels (cm^{-1}) of the isotopologue D_2 below the centrifugal barrier maxima V_b.

J	v	Energy	$V_b(cm^{-1})$	J	v	Energy	$V_b(cm^{-1})$
6	20	7.286	27.748	34	8	877.976	2021.152
7	20	38.085	40.145	34	9	1681.381	2021.152
10	19	53.958	94.620	35	7	507.176	2183.524
11	19	116.476	119.132	35	8	1447.799	2183.524
13	18	95.910	178.744	35	9	2110.247	2183.524
14	18	200.770	214.193	36	6	85.086	2354.753
15	17	32.429	253.640	36	7	1143.423	2354.753
16	17	199.828	297.264	36	8	1978.629	2354.753
17	17	334.945	345.244	37	6	781.646	2535.190
18	16	180.789	397.764	37	7	1750.354	2535.190
19	16	380.062	455.008	37	8	2445.526	2535.190
20	15	165.142	517.166	38	5	371.006	2725.210
21	15	420.374	584.430	38	6	1454.342	2725.210
22	14	165.962	656.997	38	7	2317.639	2725.210
22	15	632.076	656.997	39	5	1103.353	2925.214
23	14	473.361	735.068	39	6	2097.043	2925.214
24	13	192.314	818.850	39	7	2821.036	2925.214
24	14	742.428	818.850	40	4	705.550	3135.630
25	13	549.067	908.556	40	5	1811.173	3135.630
26	12	250.650	1004.403	40	6	2699.693	3135.630
26	13	869.176	1004.403	41	3	266.929	3356.923
27	12	654.282	1106.617	41	4	1472.346	3356.923
27	13	1106.175	1106.617	41	5	2488.576	3356.923
28	11	345.579	1215.432	41	6	3238.800	3356.923
28	12	1021.561	1215.432	42	3	1088.440	3589.595
29	11	793.797	1331.088	42	4	2214.193	3589.595
29	12	1317.394	1331.088	42	5	3125.885	3589.595
30	10	480.369	1453.836	43	2	665.230	3834.197
30	11	1205.295	1453.836	43	3	1888.553	3834.197
31	9	98.750	1583.938	43	4	2925.524	3834.197
31	10	971.035	1583.938	43	5	3700.672	3834.197
31	11	1550.071	1583.938	44	1	207.244	4091.333
32	9	657.310	1721.666	44	2	1519.320	4091.333
32	10	1424.297	1721.666	44	3	2663.582	4091.333
33	8	279.713	1867.304	44	4	3597.210	4091.333
33	9	1188.449	1867.304	45	1	1112.092	4361.677
33	10	1812.806	1867.304	45	2	2351.843	4361.677

Table S10: Continued.

J	v	Energy	$V_b(\text{cm}^{-1})$
45	3	3408.387	4361.677
45	4	4208.430	4361.677
46	0	671.248	4645.980
46	1	1997.799	4645.980
46	2	3159.476	4645.980
46	3	4114.614	4645.980
47	0	1606.906	4945.099
47	1	2862.057	4945.099
47	2	3937.633	4945.099
47	3	4763.781	4945.099
48	0	2523.477	5260.017
48	1	3701.988	5260.017
48	2	4679.020	5260.017
49	0	3419.031	5591.887
49	1	4513.716	5591.887
49	2	5368.285	5591.887
50	0	4291.231	5942.086
50	1	5291.301	5942.086
50	2	5940.639	5942.086
51	0	5137.063	6312.306
51	1	6023.213	6312.306
52	0	5952.227	6704.705
52	1	6672.643	6704.705
53	0	6729.391	7122.181
54	0	7451.384	7568.944

Table S11: Quasi-bound energy levels (cm^{-1}) of the isotopologue DT below the centrifugal barrier maxima V_b.

J	v	Energy	$V_b(cm^{-1})$	J	v	Energy	$V_b(cm^{-1})$
7	22	23.856	31.807	34	10	188.248	1584.622
10	21	15.946	75.191	34	11	975.382	1584.622
11	21	77.529	94.700	34	12	1522.840	1584.622
13	20	25.086	142.092	35	10	692.074	1710.116
14	20	124.852	170.244	35	11	1391.058	1710.116
15	20	200.335	201.544	36	9	354.686	1842.186
16	19	83.369	236.127	36	10	1172.554	1842.186
17	19	216.911	274.127	36	11	1756.923	1842.186
18	18	25.212	315.682	37	9	891.695	1981.059
18	19	315.106	315.682	37	10	1621.394	1981.059
19	18	207.478	360.930	38	8	559.530	2126.971
20	18	366.387	410.010	38	9	1404.583	2126.971
21	17	194.818	463.064	38	10	2020.686	2126.971
22	17	402.683	520.237	39	7	183.271	2280.172
23	16	191.015	581.675	39	8	1128.404	2280.172
23	17	570.189	581.675	39	9	1885.224	2280.172
24	16	443.893	647.528	40	7	803.242	2440.924
25	15	204.385	717.950	40	8	1672.475	2440.924
25	16	663.374	717.950	40	9	2316.276	2440.924
26	15	499.790	793.097	41	6	435.934	2609.503
27	14	240.962	873.129	41	7	1402.792	2609.503
27	15	764.224	873.129	41	8	2183.775	2609.503
28	14	576.849	958.212	42	5	31.585	2786.203
28	15	958.066	958.212	42	6	1086.079	2786.203
29	13	305.177	1048.513	42	7	1976.987	2786.203
29	14	882.558	1048.513	42	8	2645.389	2786.203
30	13	679.694	1144.209	43	5	728.882	2971.336
30	14	1130.254	1144.209	43	6	1715.259	2971.336
31	12	400.290	1245.478	43	7	2518.037	2971.336
31	13	1024.073	1245.478	44	4	336.088	3165.236
32	11	59.462	1352.507	44	5	1408.150	3165.236
32	12	811.708	1352.507	44	6	2318.701	3165.236
32	13	1315.477	1352.507	44	7	3009.458	3165.236
33	11	528.682	1465.489	45	4	1061.983	3368.261
33	12	1192.642	1465.489	45	5	2066.075	3368.261

Table S11: Continued.

J	v	Energy	$V_b(\text{cm}^{-1})$	J	v	Energy	$V_b(\text{cm}^{-1})$
45	6	2888.847	3368.261	55	0	4400.685	6007.826
46	3	681.470	3580.796	55	1	5315.967	6007.826
46	4	1769.471	3580.796	55	2	5970.318	6007.826
46	5	2698.086	3580.796	56	0	5173.274	6348.571
46	6	3409.770	3580.796	56	1	5996.125	6348.571
47	2	270.346	3803.262	57	0	5920.514	6708.003
47	3	1435.037	3803.262	57	1	6620.178	6708.003
47	4	2455.426	3803.262	58	0	6637.275	7088.360
47	5	3296.952	3803.262	59	0	7312.987	7492.783
48	2	1067.347	4036.115	60	0	7914.188	7926.200
48	3	2169.994	4036.115				
48	4	3115.547	4036.115				
48	5	3847.553	4036.115				
49	1	670.017	4279.857				
49	2	1847.804	4279.857				
49	3	2883.451	4279.857				
49	4	3743.051	4279.857				
50	0	246.019	4535.045				
50	1	1493.315	4535.045				
50	2	2609.644	4535.045				
50	3	3571.449	4535.045				
50	4	4323.991	4535.045				
51	0	1110.045	4802.302				
51	1	2300.026	4802.302				
51	2	3350.264	4802.302				
51	3	4227.820	4802.302				
51	4	4800.403	4802.302				
52	0	1958.963	5082.332				
52	1	3088.330	5082.332				
52	2	4066.146	5082.332				
52	3	4840.227	5082.332				
53	0	2791.446	5375.941				
53	1	3855.974	5375.941				
53	2	4751.918	5375.941				
53	3	5363.732	5375.941				
54	0	3605.965	5684.068				
54	1	4599.981	5684.068				
54	2	5397.314	5684.068				

Table S12: Quasi-bound energy levels (cm^{-1}) of the isotopologue T_2 below the centrifugal barrier maxima V_b.

J	v	Enery	$V_b(cm^{-1})$	J	v	Enery	$V_b(cm^{-1})$
6	25	9.704	16.416	34	14	482.358	1181.387
7	25	23.864	23.881	34	15	1004.851	1181.387
10	24	28.396	56.727	35	13	197.262	1273.643
11	24	67.081	71.491	35	14	828.807	1273.643
13	23	30.433	107.327	35	15	1248.216	1273.643
14	23	100.796	128.591	36	13	591.877	1370.551
15	23	152.088	152.212	36	14	1148.965	1370.551
16	22	60.126	178.287	37	12	305.463	1472.251
17	22	159.538	206.912	37	13	967.105	1472.251
18	22	236.142	238.182	37	14	1425.203	1472.251
19	21	134.883	272.196	38	12	728.284	1578.888
20	21	258.999	309.050	38	13	1315.813	1578.888
21	20	99.785	348.844	39	11	442.721	1690.609
21	21	348.713	348.844	39	12	1131.156	1690.609
22	20	263.423	391.678	39	13	1622.005	1690.609
23	19	65.314	437.652	40	10	116.871	1807.570
23	20	404.672	437.652	40	11	892.692	1807.570
24	19	265.971	486.870	40	12	1507.158	1807.570
25	18	39.274	539.436	41	10	609.797	1929.930
25	19	447.410	539.436	41	11	1322.161	1929.930
26	18	275.390	595.456	41	12	1841.285	1929.930
26	19	590.096	595.456	42	9	288.511	2057.858
27	17	27.501	655.036	42	10	1085.930	2057.858
27	18	493.369	655.036	42	11	1724.362	2057.858
28	17	297.755	718.288	43	9	807.222	2191.528
28	18	680.512	718.288	43	10	1541.044	2191.528
29	16	34.428	785.325	43	11	2084.917	2191.528
29	17	550.136	785.325	44	8	491.756	2331.123
30	16	337.572	856.260	44	9	1308.608	2331.123
30	17	774.612	856.260	44	10	1968.500	2331.123
31	15	63.428	931.212	45	7	143.795	2476.834
31	16	622.705	931.212	45	8	1035.356	2476.834
32	15	398.244	1010.301	45	9	1788.522	2476.834
32	16	881.136	1010.301	45	10	2354.345	2476.834
33	14	117.063	1093.650	46	7	726.753	2628.862
33	15	714.693	1093.650	46	8	1561.175	2628.862
33	16	1086.680	1093.650	46	9	2240.440	2628.862

Table S12: Continued.

J	v	Energy	$V_b(\mathrm{cm}^{-1})$	J	v	Energy	$V_b(\mathrm{cm}^{-1})$
47	6	386.879	2787.421	56	0	231.013	4550.876
47	7	1294.429	2787.421	56	1	1355.799	4550.876
47	8	2065.158	2787.421	56	2	2377.608	4550.876
47	9	2650.744	2787.421	56	3	3281.526	4550.876
48	5	18.980	2952.735	56	4	4039.871	4550.876
48	6	993.548	2952.735	56	5	4546.318	4550.876
48	7	1843.963	2952.735	57	0	1006.686	4790.121
48	8	2540.909	2952.735	57	1	2086.128	4790.121
49	5	662.466	3125.042	57	2	3056.900	4790.121
49	6	1584.579	3125.042	57	3	3899.618	4790.121
49	7	2371.407	3125.042	57	4	4570.576	4790.121
49	8	2975.136	3125.042	58	0	1770.414	5039.568
50	4	304.308	3304.595	58	1	2802.315	5039.568
50	5	1292.118	3304.595	58	2	3718.283	5039.568
50	6	2157.216	3304.595	58	3	4491.537	5039.568
50	7	2870.532	3304.595	58	4	5026.073	5039.568
51	4	970.392	3491.667	59	0	2521.281	5299.785
51	5	1905.880	3491.667	59	1	3502.941	5299.785
51	6	2707.653	3491.667	59	2	4359.015	5299.785
51	7	3328.447	3491.667	59	3	5048.104	5299.785
52	3	622.428	3686.548	60	0	3258.259	5571.424
52	4	1622.389	3686.548	60	1	4186.247	5571.424
52	5	2501.127	3686.548	60	2	4974.974	5571.424
52	6	3229.880	3686.548	60	3	5540.212	5571.424
53	2	250.722	3889.553	61	0	3980.155	5855.240
53	3	1310.457	3889.553	61	1	4849.926	5855.240
53	4	2258.364	3889.553	61	2	5558.609	5855.240
53	5	3074.231	3889.553	62	0	4685.541	6152.123
53	6	3711.557	3889.553	62	1	5490.635	6152.123
54	2	973.031	4101.022	62	2	6089.036	6152.123
54	3	1984.258	4101.022	63	0	5372.611	6463.150
54	4	2875.851	4101.022	63	1	6102.663	6463.150
54	5	3619.487	4101.022	64	0	6038.910	6789.662
54	6	4100.206	4101.022	64	1	6672.569	6789.662
55	1	612.534	4321.326	65	0	6680.660	7133.396
55	2	1682.444	4321.326	66	0	7290.618	7496.743
55	3	2642.038	4321.326	67	0	7848.663	7883.312
55	4	3471.453	4321.326				
55	5	4125.324	4321.326				

Table S13: Values of S° ($J\,K^{-1}mol^{-1}$) for the main isotopologue H_2 calculated using different methods.

T (K)	Popovas-Jorgensen[a]	Present-bl	JANAF[b]	Present-qbl	LeRoy[c]	Present-qbl
10	35.5033(0.004)	35.5018		35.502	47.0284(0.001)	47.0281
50	77.6385(0.007)	77.6331		77.633	89.1608(0.002)	89.1594
100	100.7201(0.003)	100.7269	100.727(0.000)	100.727	112.2543(0.001)	112.2532
150	111.6678(0.002)	111.6651		111.665	123.1925(0.001)	123.1914
200	119.4144(0.002)	119.4118	119.412(0.000)	119.412	130.9392(0.001)	130.9380
250	125.6425(0.002)	125.6397	125.640(0.000)	125.640	137.1673(0.001)	137.1660
300	130.8804(0.002)	130.8576	130.858(0.000)	130.858	142.3852(0.001)	142.3839
400	139.2179(0.002)	139.2152	139.216(0.001)	139.215	150.7429(0.001)	150.7415
500	145.7392(0.002)	145.7366	145.737(0.000)	145.737	157.2643(0.001)	157.2629
600	151.0793(0.002)	151.0768	151.077(0.000)	151.077	162.6046(0.001)	162.6031
700	155.6077(0.002)	155.6053	155.606(0.001)	155.605	167.1331(0.001)	167.1316
800	159.5499(0.001)	159.5476	159.548(0.000)	159.548	171.0755(0.001)	171.0739
900	163.0531(0.001)	163.0509	163.051(0.000)	163.051	174.5788(0.001)	174.5771
1000	166.2175(0.001)	166.2153	166.216(0.001)	166.215	177.7433(0.001)	177.7416
2000	188.4192(0.001)	188.4165	188.418(0.001)	188.417	199.9455(0.001)	199.9428
3000	202.8903(0.002)	202.8871	202.881(0.002)	202.887	214.4168(0.002)	214.4135
4000	213.8379(0.002)	213.8345	213.848(0.004)	213.839	225.3691(0.002)	225.3656
5000	222.7186(0.002)	222.7151	222.767(0.003)	222.755	234.2832(0.001)	234.2809
6000	230.1763(0.002)	230.1726	230.323(0.005)	230.315	241.8399(-0.001)	241.8411
7000	236.5055(0.002)	236.5016		236.826	248.3449(-0.003)	248.3525
8000	241.8788(0.002)	241.8748		242.438	253.9485(-0.006)	253.9642
9000	246.4418(0.002)	246.4369		247.259	258.7613(-0.009)	258.7854
10000	250.3329(0.003)	250.3247		251.398	262.8924(-0.012)	262.9242

[a] A. Popovas and U. G. Jorgensen, A&A, **595**, A130 (2016)
[b] M. W. Chase, Journal of Physical and Chemical Reference Data, Monograph, No. 9 (1998)
[c] R. J. Le Roy *et al*, J. Phys. Chem., **94**, 923 (1990)

Table S14: Values of H°- H°($T_{ref.}$) (kJ mol^{-1}) for the main isotopologue H_2 calculated using different methods.

T (K)	Popovas-Jorgensen[a]	Present-bl	JANAF[b]	Present-qbl	LeRoy[c]	Present-qbl
10	0.2079(0.000)	0.2079		-8.259	0.2079(0.000)	0.2079
50	1.3650(0.007)	1.3649		-7.102	1.3649(0.007)	1.3649
100	2.9991(-0.003)	2.9992	-5.468(0.000)	-5.468	2.9992(0.000)	2.9992
150	4.3453(-0.002)	4.3454		-4.122	4.3454(0.000)	4.3454
200	5.6928(-0.002)	5.6929	-2.774(0.000)	-2.774	5.6929(0.000)	5.6929
250	7.0892(-0.001)	7.0893	-1.378(0.000)	-1.378	7.0893(0.000)	7.0893
300	8.5205(-0.001)	8.5206	0.053(0.000)	0.053	8.5207(0.001)	8.5206
400	11.4264(-0.001)	11.4265	2.959(0.000)	2.959	11.4266(0.001)	11.4265
500	14.3490(-0.001)	14.3492	5.882(0.000)	5.882	14.3493(0.001)	14.3492
600	17.2781(-0.001)	17.2783	8.111(0.000)	8.111	17.2784(0.001)	17.2783
700	20.2158(-0.002)	20.2161	11.749(0.000)	11.749	20.2163(0.001)	20.2161
800	23.1683(-0.002)	23.1687	14.702(0.007)	14.701	23.1689(0.001)	23.1687
900	26.1429(-0.002)	26.1433	17.676(0.000)	17.676	26.1435(0.001)	26.1433
1000	29.1465(-0.002)	29.1470	20.680(0.000)	20.680	29.1473(0.001)	29.1470
2000	61.4169(0.002)	61.4165	52.951(0.004)	52.949	61.4183(0.003)	61.4165
3000	97.2012(0.001)	97.1998	88.740(0.008)	88.733	97.2033(0.003)	97.2001
4000	135.3028(0.002)	135.3006	126.874(0.018)	126.851	135.3222(0.003)	135.3184
5000	175.1286(0.002)	175.1255	166.876(0.024)	166.836	175.3023(-0.001)	175.3035
6000	216.0429(0.002)	216.0386	208.341(0.010)	208.321	216.7671(-0.010)	216.7886
7000	257.0964(0.002)	257.0914		250.565	258.9795(-0.020)	259.0326
8000	297.3227(0.002)	297.3163		292.582	300.9261(-0.041)	301.0497
9000	336.0468(0.004)	336.0328		333.501	341.7737(-0.057)	341.9687
10000	372.9614(0.012)	372.9163		372.768	380.9629(-0.070)	381.2355

[a]A. Popovas and U. G. Jorgensen, A&A, **595**, A130 (2016)
[b]M. W. Chase, Journal of Physical and Chemical Reference Data, Monograph, No. 9 (1998)
[c]R. J. Le Roy *et al*, J. Phys. Chem., **94**, 923 (1990)

Table S15: Values of -(G°- H°($T_{ref.}$))/T ($J\,K^{-1}mol^{-1}$) for the main isotopologue H_2 calculated using different methods.

T (K)	Popovas-Jorgensen[a]	Present-bl	JANAF[b]	Present-qbl	LeRoy[c]	Present-qbl
10	14.7171(0.010)	14.7156		861.440	26.2421(0.001)	26.2418
50	50.3390(0.006)	50.3360		219.681	61.8631(0.001)	61.8623
100	70.7389(0.005)	70.7353	155.408(0.000)	155.408	82.2626(0.001)	82.2616
150	82.6989(0.004)	82.6956		139.144	94.2229(0.001)	94.2219
200	90.9503(0.003)	90.9472	133.284(0.001)	133.283	102.4745(0.001)	102.4734
250	97.2857(0.003)	97.2827	131.152(0.000)	131.152	108.8100(0.001)	108.8089
300	102.4586(0.003)	102.4556	130.680(0.000)	130.680	113.9830(0.001)	113.9819
400	110.6519(0.003)	110.6489	131.817(0.000)	131.817	122.1764(0.001)	122.1752
500	117.0411(0.003)	117.0382	133.973(0.000)	133.973	128.5658(0.001)	128.5645
600	122.2825(0.002)	122.2797	136.392(0.000)	136.392	133.8072(0.001)	133.8060
700	126.7279(0.002)	126.7251	138.822(0.001)	138.821	138.2527(0.001)	138.2514
800	130.5895(0.002)	130.5867	141.171(0.000)	141.171	142.1144(0.001)	142.1130
900	134.0055(0.002)	134.0028	143.411(0.000)	143.411	145.5305(0.001)	145.5291
1000	137.0709(0.002)	137.0683	145.536(0.000)	145.536	148.5960(0.001)	148.5946
2000	157.7107(0.002)	157.7082	161.943(0.001)	161.942	169.2363(0.001)	169.2345
3000	170.4898(0.002)	170.4872	173.311(0.001)	173.310	182.0175(0.001)	182.0135
4000	180.0122(0.002)	180.0094	182.129(0.001)	182.127	191.5385(0.001)	191.5360
5000	187.6929(0.002)	187.6900	189.392(0.003)	189.387	199.2228(0.001)	199.2202
6000	194.1692(0.002)	194.1661	195.600(0.003)	195.595	205.7120(0.001)	205.7097
7000	199.7774(0.002)	199.7743		201.031	211.3492(0.001)	211.3479
8000	204.7135(0.002)	204.7102		205.864	216.3327(-0.000)	216.3330
9000	209.1033(0.002)	209.0999		210.203	220.7864(-0.001)	220.7889
10000	213.0368(0.002)	213.0331		214.121	224.7957(-0.002)	224.8007

[a] A. Popovas and U. G. Jorgensen, A&A, **595**, A130 (2016)
[b] M. W. Chase, Journal of Physical and Chemical Reference Data, Monograph, No. 9 (1998)
[c] R. J. Le Roy *et al*, J. Phys. Chem., **94**, 923 (1990)

Table S16: Thermodynamic quantities of the isotopologue D_2 for equilibrium and normal mixtures calculated by the method of Colonna *et al.* (see text), using the rovibrational energy levels extracted from the adiabatic Pachoucki and Komasa potentials and including the quasi-bound levels.

	C°_P		S°		H°- $H^\circ(0)$		-(G°- $H^\circ(0)$)/T	
T (K)	Normal	Equilibrio	Normal	Equilibrio	Normal	Equilibrio	Normal	Equilibrio
5	20.7862	20.7863	62.5688	56.1528	0.342313	0.103931	-5.89376	35.3666
10	20.7862	20.9557	76.9767	70.5827	0.446244	0.208059	32.3523	49.7768
20	20.7979	23.8075	91.3855	85.8502	0.654123	0.430006	58.6793	64.3499
30	21.1671	26.2298	99.8621	96.0175	0.863298	0.681580	71.0855	73.2981
40	22.6076	27.7163	106.121	103.774	1.08119	0.951546	79.0909	79.9851
50	24.8970	29.0288	111.403	110.103	1.31835	1.23544	85.0361	85.3944
60	27.1660	30.0050	116.149	115.489	1.57902	1.53098	89.8324	89.9725
70	28.8096	30.5182	120.470	120.158	1.85953	1.83397	93.9055	93.9588
80	29.7192	30.6458	124.384	124.245	2.15273	2.14005	97.4749	97.4947
90	30.0723	30.5364	127.909	127.850	2.45205	2.44610	100.664	100.671
100	30.0996	30.3184	131.081	131.057	2.75310	2.75042	103.550	103.553
150	29.4126	29.4156	143.150	143.150	4.24020	4.24017	114.882	114.882
200	29.2054	29.2054	151.575	151.575	5.70410	5.70410	123.054	123.054
250	29.1858	29.1858	158.088	158.088	7.16361	7.16361	129.434	129.434
300	29.1961	29.1961	163.410	163.410	8.62312	8.62312	134.667	134.667
400	29.2438	29.2438	171.815	171.815	11.5447	11.5447	142.953	142.953
500	29.3683	29.3683	178.352	178.352	14.4744	14.4744	149.403	149.403
600	29.6217	29.6217	183.727	183.727	17.4228	17.4228	154.689	154.689

Table S17: Thermodynamic quantities of the isotopologue T_2 for equilibrium mixtures and normal mixtures calculated by the method of Colonna *et al.* (see text), using the rovibrational energy levels extracted from the adiabatic Pachoucki and Komasa potentials and including the quasi-bound levels.

	C_P°		S°		H°- $H^\circ(0)$		-(G°- $H^\circ(0)$)/T	
T (K)	Normal	Equilibrio	Normal	Equilibrio	Normal	Equilibrio	Normal	Equilibrio
5	20.7862	20.8841	64.6681	46.3002	0.463378	0.103973	-28.0075	25.5056
10	20.7863	28.1656	79.0760	62.2468	0.567309	0.221025	22.3451	40.1443
20	20.9252	36.4559	93.5017	86.5753	0.775491	0.577112	54.7272	57.7197
30	21.9328	29.5619	102.141	99.9333	0.988900	0.904164	69.1777	69.7945
40	23.6254	26.9089	108.677	107.993	1.21648	1.18370	78.2644	78.4007
50	25.2415	26.5040	114.128	113.929	1.46108	1.44965	84.9062	84.9363
60	26.4951	26.9263	118.846	118.793	1.72007	1.71646	90.1784	90.1850
70	27.4089	27.5425	123.003	122.989	1.98985	1.98879	94.5767	94.5781
80	28.0515	28.0899	126.708	126.704	2.26735	2.26706	98.3658	98.3661
90	28.4832	28.4937	130.038	130.038	2.55017	2.55010	101.703	101.703
100	28.7592	28.7619	133.055	133.055	2.83650	2.83648	104.690	104.690
150	29.1346	29.1346	144.818	144.818	4.28768	4.28768	116.234	116.234
200	29.1637	29.1637	153.205	153.205	5.74533	5.74533	124.478	124.478
250	29.1788	29.1788	159.714	159.714	7.20388	7.20388	130.898	130.898
300	29.2005	29.2005	165.036	165.036	8.66332	8.66332	136.158	136.158
400	29.3160	29.3160	173.449	173.449	11.5880	11.5880	144.479	144.479
500	29.6098	29.6098	180.018	180.018	14.5326	14.5326	150.953	150.953
600	30.0982	30.0982	185.457	185.457	17.5165	17.5165	156.263	156.263
